# The subcellular architecture of *Paratrypanosoma confusum* revealed by CryoET: A window into early trypanosome evolution

**DOI:** 10.1073/pnas.2521233122

**Published:** 2025-12-08

**Authors:** Carolina de Lima Alcantara, Matthias Pöge, Wolfgang Baumeister, Juergen M. Plitzko, Wanderley de Souza

**Affiliations:** ^a^Instituto de Biofísica Carlos Chagas Filho, Centro de Pesquisas em Medicina de Precisão, Universidade Federal do Rio de Janeiro, Rio de Janeiro 21941-904, Brazil; ^b^Research Group Molecular Structural Biology, Max Planck Institute of Biochemistry, Martinsried 82152, Germany; ^c^Research Group CryoEM Technology, Max Planck Institute of Biochemistry, Martinsried 82152, Germany; ^d^Centro Nacional de Biologia Estrutural e Bioimagem e Instituto Nacional de Ciência e Tecnologia em Biologia Estrutural e Bioimagens, Universidade Federal do Rio de Janeiro, Rio de Janeiro 21941-902, Brazil; ^e^Centro Multiusuário para Análise de Fenômenos Biomédicos, Universidade do Estado do Amazonas, Manaus 21941-902, Brazil

**Keywords:** cryo-ET, trypanosomatids, ultrastructure, cryo FIB-SEM

## Abstract

The trypanosomatid *Paratrypanosoma confusum* represents a key evolutionary link between free-living bodonids and parasitic trypanosomes, yet its cellular architecture remains poorly characterized. Here, we used cryofocused ion beam scanning electron microscopy to prepare lamellae for cryoelectron tomography, enabling nanometer-scale imaging of its ultrastructure in near-native conditions. Our analysis revealed structures never before described in trypanosomatids, including an elaborate cytoskeletal network surrounding the flagellar pocket (FP), desmosome-like connections linking the FP membrane to the flagellum, and a cytopharyngeal-like invagination arising from the pocket. Luminal particles inside the FP were reconstructed in 3D, providing insight into their organization and potential role. The contractile vacuole complex was characterized by two bladders with distinct surface protein coats. Organelles of the biosynthetic and secretory pathways were also resolved, including the Golgi complex, ER exit sites, multivesicular bodies, and a novel multivesicular tubule. We also identified large, autophagosome-like organelles containing intraluminal vesicles and hemifused vesicles, structurally resembling hemifusomes recently described in mammalian cells. These structures, along with periodic striations observed in lipid-rich compartments, suggest conserved mechanisms of vesicle biogenesis. Finally, we describe the nuclear architecture, including well-preserved nuclear pores with defined subcomplexes, chromatin fibers resembling nucleosome arrays, and putative nuclear envelope–chromatin linkages. Together, these findings highlight the cellular complexity of *P. confusum*, providing insights into the evolution of cellular architecture within trypanosomatids and underscoring the potential of this organism as a model for high-resolution structural studies.

## Significance

*Paratrypanosoma confusum* is the earliest-diverging lineage of trypanosomatids, a group that includes several important human and veterinary parasites. Despite its phylogenetic position, little was known about its cellular organization. Using cryoelectron tomography (cryo-ET), we mapped the three-dimensional architecture of *P. confusum,* revealing a unique combination of structures conserved across kinetoplastids and others potentially ancestral. Our work establishes *P. confusum* as a powerful model for investigating early eukaryotic evolution and highlights the transformative potential of cryo-ET in protist cell biology. By visualizing the organization of key organelles in this understudied protist, our study contributes to a broader understanding of eukaryotic cell diversity and evolution.

The family Trypanosomatidae comprises a group of uniflagellate protozoan parasites, with some members being responsible for a multitude of neglected tropical diseases such as *Trypanosoma cruzi* (Chagas disease), *Trypanosoma brucei* (sleeping sickness), and *Leishmania* sp. (Leishmaniasis), inflicting immense suffering on humans and animals. Understanding the intricate biology of these parasites is paramount for developing effective control strategies against these debilitating illnesses. However, the evolutionary history of the trypanosomatids remains poorly understood, hindering our ability to predict future parasite emergence and to develop targeted interventions.

In 2013, Julius Lukes group described a new species of monoxenic trypanosomatid, isolated from the gut of mosquitoes from Cullex genus (1). The new genus *Paratrypanosoma* stands as a critical missing link in the evolutionary puzzle of trypanosomatids. Its sole member, *Paratrypanosoma confusum*, exhibits characteristics of both free-living bodonids and parasitic trypanosomes, potentially bridging the gap between these two divergent lifestyles. Previous studies using scanning and transmission electron microscopy (TEM) of fixed, resin-embedded cells provided an overview of *P. confusum’s* cellular organization, suggesting that it might represent an ancestral form of parasitic trypanosomes (2). However, the limitations of conventional electron microscopy methods have hampered our ability to fully understand *P. confusum’s* biology, its ecological role, and its evolutionary relationship to parasitic trypanosomes.

Conventional TEM of thin sections has been intensely used to describe in detail the structural organization of pathogenic and nonpathogenic trypanosomatids (3, 4). Studies carried out in several cell types have shown cryofixation followed by Cryo-FIB milling to obtain cryolamellae and imaging by Cryo-ET allows for a much better preservation of cell structure revealing new relevant information on cell organization (5–7). Here, we make a description of the structural organization of a trypanosomatid using this unique approach, opening windows into the molecular architecture of *P. confusum* in a near-native state. Our comprehensive examination of different cell organelles reveals interesting features, including the presence of an elaborated contractile vacuole (CV), a complex filamentous network at the flagellar pocket (FP), the detailed architecture of the cytostome–cytopharynx complex and other biosynthetic-secretory organelles, the presence of a dense surface coat within the FP, the ultrastructural organization of nucleus, kinetoplast, glycosomes and acidocalcisomes. These findings provide a deeper understanding of *P. confusum*’s unique adaptations, potentially shedding light on the evolutionary trajectory of trypanosomes and offering valuable insights for understanding the evolution of parasitism within the trypanosome lineage.

## Results and Discussion

In axenic culture, *P. confusum* exhibits three main morphotypes. Promastigotes and choanomastigotes are liberform, with the flagellum exiting the FP without attachment to the cell body (2). Haptomonads attach to the culture flask via a spread flagellum, remaining attached to the substrate (2). For our analysis, only detached parasites were used, most of them being promastigote forms.

In situ cryoelectron tomography (cryo-ET) was performed in thin cryolamellae produced by Cryo-FIB milling of vitrified parasites. An overview of the process is depicted in *SI Appendix*, Fig. S1. We acquired over a hundred tomograms from various regions of the cell, enabling a comprehensive analysis of *P. confusum* cell biology. We were able to visualize organelles and structural features with nanometer resolution, revealing unprecedented details never described in trypanosomatids.

### FP Architecture.

#### Distinct luminal particles inside the FP of *P. confusum*.

**Fig. 1.**
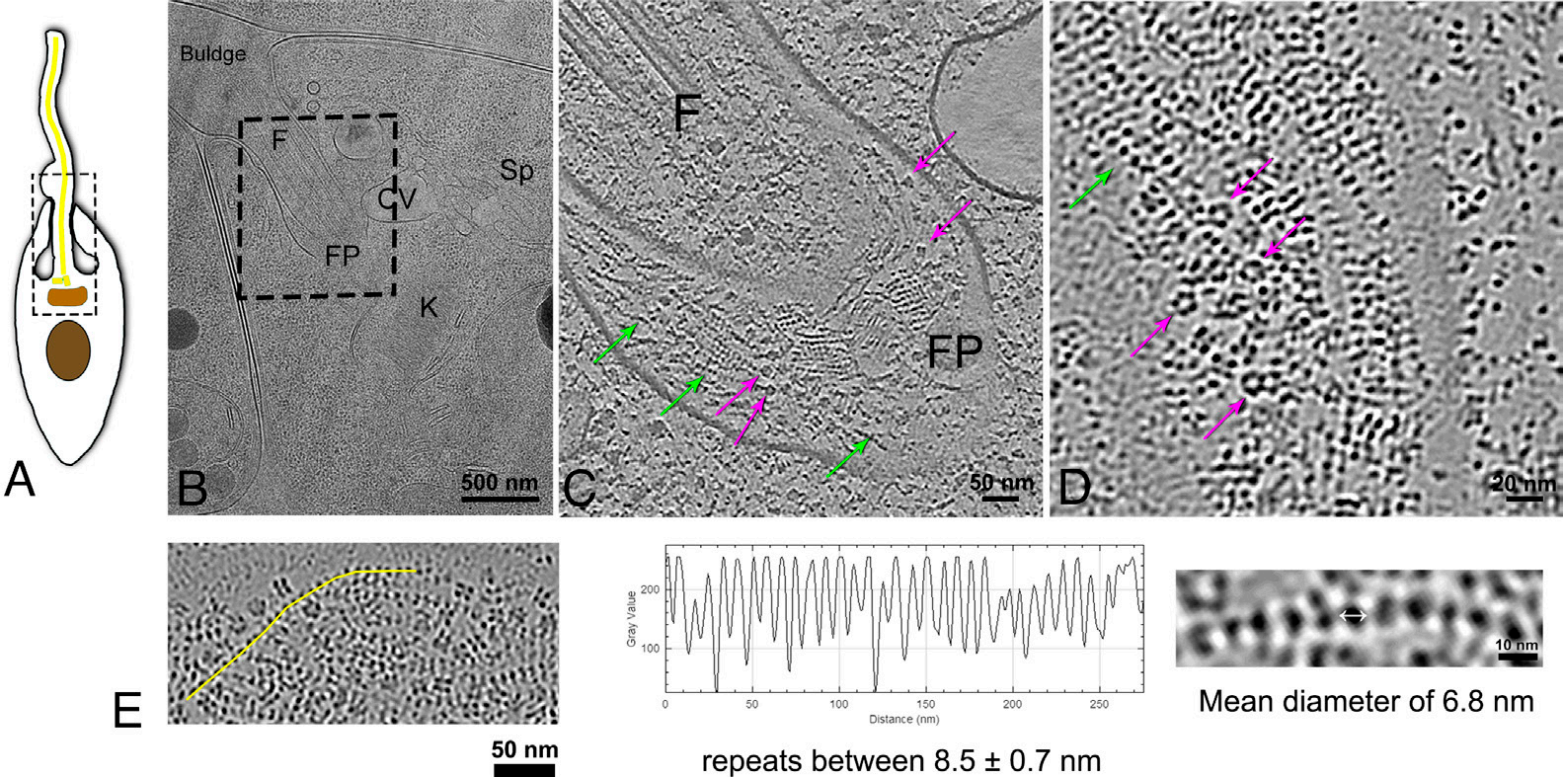
Identification and characterization of luminal particles in the FP of *P. confusum*. (*A*) Schematic representation of the cell illustrating its main organelles and cellular structures (nucleus-brown; kinetoplast-orange; basal body (BB)/flagellar axoneme-yellow). The highlighted area corresponds to the region where tomogram shown in *B* was acquired. (*B*) TEM image of the lamella from the region of interest (*A*), depicting key organelles such as the flagellum (F), CV, spongiome (Sp), kinetoplast (K), and FP. The hatched rectangle indicates the area where the cryotomogram was acquired. (*C*) Tomographic slice corresponding to the rectangle region in panel b, highlighting the lumen of the FP filled with distinct particles. Violet arrows indicate five-lobed particles, while light green arrows point to filaments of bead-like particles. (*D*) Higher magnification tomographic slice of a different tomogram showing the FP lumen filled with particles showing the two types of particles already mentioned in (*C*). (*E*) Tomographic slice showing an example region where a line scan (yellow line) through the bead filaments was measured. The concatenated beads are organized periodically in repeats of 8.5 ± 0.7 nm measured by line scan plot of the tomograms. The beads mean diameter was 6.8 nm.

Multiple tomograms of *P. confusum* FP area (n = 10 tomograms) revealed a luminal content consistently filled with distinct particles (Fig. 1). These particles could be morphologically classified in cylindrical five-lobed particles and filaments of bead-like particles (Fig. 1 *C* and *D*). The cylindrical particles were found scattered between the bead-like filaments, which were the most abundant types of particles found inside FP. Bead-like filaments (Fig. 1*E*) were shown to be formed by periodically spaced beads as analyzed by line scan plot of tomogram-derived images of the filaments, and a consistent diameter of 6.8 nm accessed by measuring averaged tomograms (Fig. 1*E*).

When we compare TEM data of the FP lumen in conventional, chemically fixed, and dehydrated cells with those obtained on vitrified cells, the preservation provided by cryofixation becomes clear. In conventional samples, the FP pocket lumen appears empty or partially filled with a low-density indistinguishable content (*SI Appendix*, Fig. S2). Only in some cells, a highly dense surface coat composed of long fibrous material and electron-dense particles (*SI Appendix*, Fig. S2*C*), which to a certain extent resemble the particles described here by cryo-ET.

An amorphous content in the FP lumen of some monoxenic and heteroxenic trypanosomatids, like promastigote forms of Leishmania sp., has already been described (8, 9). Recently, the secretion of extracellular polymeric substances (EPS) by *Lotmaria passim*, a trypanosomatid that infects honeybees, was reported (10). These works relied on conventional electron microscopy analysis, showing the presence of long interconnected bead-like particles, similar to the particles that we described here. The secretion of the EPS in *L. passim* led to the formation of biofilms and was correlated with an increased resistance in oxidative and osmotic stress. While the molecular identity and function of such intraluminal components have been better characterized in some dixenous species, the extent and structural detail of these materials may be underestimated due to limitations of conventional sample preparation techniques. In our study, the use of cryofixation and cryo-ET allowed the visualization of well-preserved, filamentous particles with a bead-like appearance, anchored to the membrane and extending into the lumen, features not previously resolved at this level of detail in any trypanosomatid.

Rather than suggesting a fundamental contrast in secretory capacity, our findings underscore the importance of high-resolution structural approaches for revealing hidden complexity in the FP across trypanosomatids. The structural preservation achieved here suggests that comparable material may be present in other species but lost or distorted in traditional EM workflows. While the biochemical nature of these filaments remains unknown in *P. confusum*, their organization and abundance raise the possibility of functional relevance, potentially in extracellular matrix formation or environmental sensing. Further molecular characterization will be required to clarify these roles.

### FP-Associated Cytoskeleton.

**Fig. 2.**
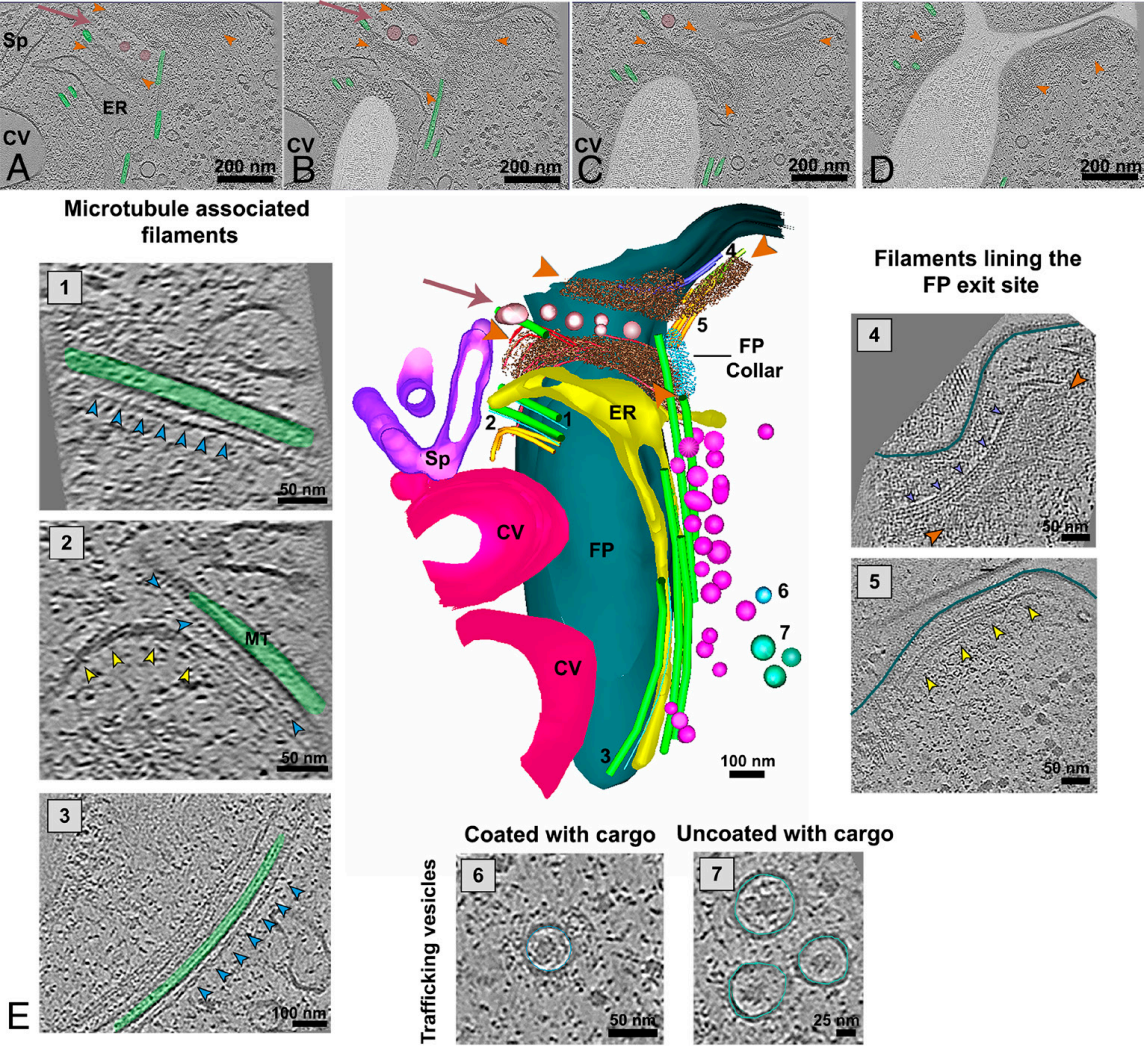
Cytoskeletal organization surrounding the FP of *P. confusum*. (*A*–*D*) Consecutive tomogram slices at different Z-planes showing cytoskeletal filaments (indicated by orange arrowheads) positioned just below the FP membrane at the flagellum exit site. The filaments are followed by a row of vesicles (highlighted in pink) and a second set of cytoskeletal filaments (also indicated by orange arrowheads). MTs associated with the FP are colored green, and the lumen of the FP is shaded in semitransparent white. Panels (*A*–*D*) illustrate how the proximal filaments appear to encircle the region around the flagellum exit, enveloping the FP at this site. (*E*) 3D rendered image of structures in the FP region manually annotated in tomogram slices. The FP membrane is depicted in cyan, and the proximal filaments surrounding the flagellum exit are marked by orange arrowheads. The vesicles located near these filaments are shown in pink (pink arrows), followed by a second set of filaments marked by additional orange arrowheads. Numerous MTs (green tubes) are associated with the FP membrane, while vesicles (lilac) are distributed along these MTs from the anterior to the posterior region. The numbered regions of interest (ROIs) correspond to specific structures observed in different slices of the tomogram: 1: Tomogram image showing a MT (green) associated with a row of filamentous proteins arranged in a dotted pattern (indicated by blue arrowheads). 2: Tomogram slice showing another MT (green) near 1, featuring the same type of filaments (blue arrowheads) and additional filaments associated with the MT (indicated by yellow arrowheads). 3: Lateral view of a MT running from the anterior to the posterior region of the parasite, showing associated filaments highlighted by blue arrowheads. 4: Filaments associated with the FP membrane at the flagellum exit, forming a braided or helical structure (marked by purple arrowheads). The FP membrane is outlined in green. 5: Additional filaments located below the FP membrane at the flagellum exit site, marked by yellow arrowheads. 6: Coated vesicles with an internal cargo. The vesicle membrane is delineated in blue. 7: Uncoated vesicles containing a cargo, suggesting a role in intracellular trafficking. The vesicle membranes are delineated in light green. Other structures shown in the model are endoplasmic reticulum (ER—yellow); CV—red purple; and spongiome (Sp—purple).

From the cytosolic point of view, the FP membrane of *P. confusum* was shown to be associated with several cytoskeleton elements. At the FP distal region, close to the flagellum exit point, known as FP neck, a network of longitudinal fibers was found to be organized around the pocket, forming a collar structure that resembles what has been already shown in other trypanosomatids (11). Two collar structures could be identified: one more distal and one more proximal in relation to the FP exit site (Fig. 2). A row of vesicles was found to separate these two collars (Fig. 2 *A*–*D*). This organization was consistently found in all the tomograms that comprised this region of the FP (n = 4) as can be seen also in another tomogram in *SI Appendix*, Fig. S3. In other trypanosomatids, the collar is an important structural feature and is crucial for the biogenesis of the FP. In *T. brucei*, Bilbo1 is the main protein responsible for the formation of the collar and the FP architecture (12). Bilbo1 is conserved across the trypanosomatid lineage, and a putative ortholog of BILBO1 (PCON_0057580) is indeed annotated in the *P. confusum* genome. However, functional validation experiments such as gene tagging, immunolabeling, or mutagenesis are still required to establish the presence and role of this or other collar-associated proteins in this organism.

Besides the collar filaments, our attention was attracted to MTs surrounding the FP (Fig. 2 and *SI Appendix*, Fig. S3). These cytosolic MTs showed different orientation relative to the FP anteroposterior axis, with some of them aligned longitudinally to the FP membrane, where they associated with coated or uncoated cargo-containing vesicles and ER that were present at the FP surroundings (Fig. 2*e*).

The first complete characterization of the FP of a trypanosomatid was made in *T. brucei* and highlighted the organization of a MT quartet (MtQ), a conserved set of MTs that originated at the BB and turned around the pocket inserting in between the subpellicular MTs at the flagellar attachment zone (FAZ) region (13). In *T. brucei*, the MtQ are the only FP-associated MTs, which contrast with the organization observed in *T. cruzi* and Leishmania. In *T. cruzi*, in addition to the MtQ, another MT quartet is associated with the cytostome–cytopharynx complex, a structure dedicated to endocytosis, as well as three additional MTs that are connected to the kinetoplast and Golgi complex (GC) (14). In *Leishmania mexicana*, besides the MtQ, two MTs are closely associated with the FP, and two additional cytoplasmic MTs have also been described, contributing to its unique FP cytoskeletal organization (15). In *P. confusum*, the first ultrastructural characterization identified the presence of the conserved MtQ, reinforcing the idea that this set of MTs plays a fundamental role in FP organization across trypanosomatids (2). The presence of the MtQ in *P. confusum* is consistent with observations in free-living bodonids, where a similar MT array surrounds the pocket (16), further supporting the evolutionary conservation of this structure. However, our data reveal a greater number of MTs surrounding the FP in *P. confusum* (approximately 8, in addition to the MtQ), compared to *T. brucei*, which contains only the MtQ (13); *T. cruzi*, which has the MtQ, the cytopharynx-associated MT quartet, and two cytoplasmic MTs (14); and Leishmania, where two pocket-associated and two cytoplasmic MTs have been described in addition to the MtQ (15). This suggests a more elaborate cytoskeletal framework in *P. confusum,* potentially linked to enhanced structural stability, vesicular trafficking, or more complex interactions with the extracellular environment. Indeed, the cytoskeletal framework surrounding the FP described here is the most complex ever reported for a trypanosomatid. This increased complexity in *P. confusum* may reflect a genuine evolutionary adaptation or, alternatively, may be attributed to the lack of well-preserved ultrastructural preparations for the other trypanosomatids examined, as conventional EM preparations were used.

**Fig. 3.**
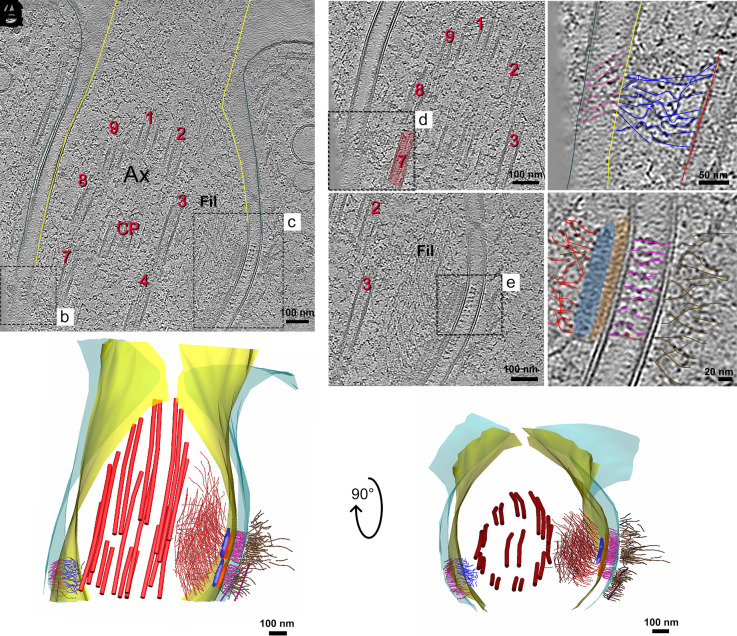
Desmosome-like structures between FP membrane and flagellar membrane. (*A*) Tomogram slice showing a transverse section near the FP exit. The flagellum membrane is outlined in yellow, while the FP membrane is outlined in cyan. The axoneme is labeled as AX, with numbered doublets of MTs (red) following the conventional arrangement, and the central pair (CP) marked. Two ROIs, *B* and *C*, are shown in higher mag in their respective panels. (*B*) Shows the adhesion region between the FP membrane and the flagellar membrane. (*C*) Detailing the structural organization within the junction. (*D*) Higher magnification of the region highlighted in *B* reveals a network of filaments (blue) linking the flagellar membrane to MT doublet 7 on the axonemal side. Intermembrane proteins connect the flagellar membrane directly with the FP membrane (pink). (*E*) Higher magnification of the region highlighted in *C* shows electron-dense regions resembling protein plaques (orange and blue) are observed adjacent to the flagellar membrane. These plaques are associated with filaments (red) extending parallel to the axoneme on the flagellar side. In the intermembrane space, pink-colored intermembrane proteins connect the FP membrane to the flagellar membrane. On the cytosolic side of the FP membrane, additional filaments (brown) are present. (*F*) Rendered image of manually annotated structures, illustrating the three-dimensional organization of the desmosome-like junction observed between the FP and flagellar membranes. The *Left* view shows a frontal perspective; the *Right* view displays the arrangement from the anterior to the FP exit.

**Fig. 4.**
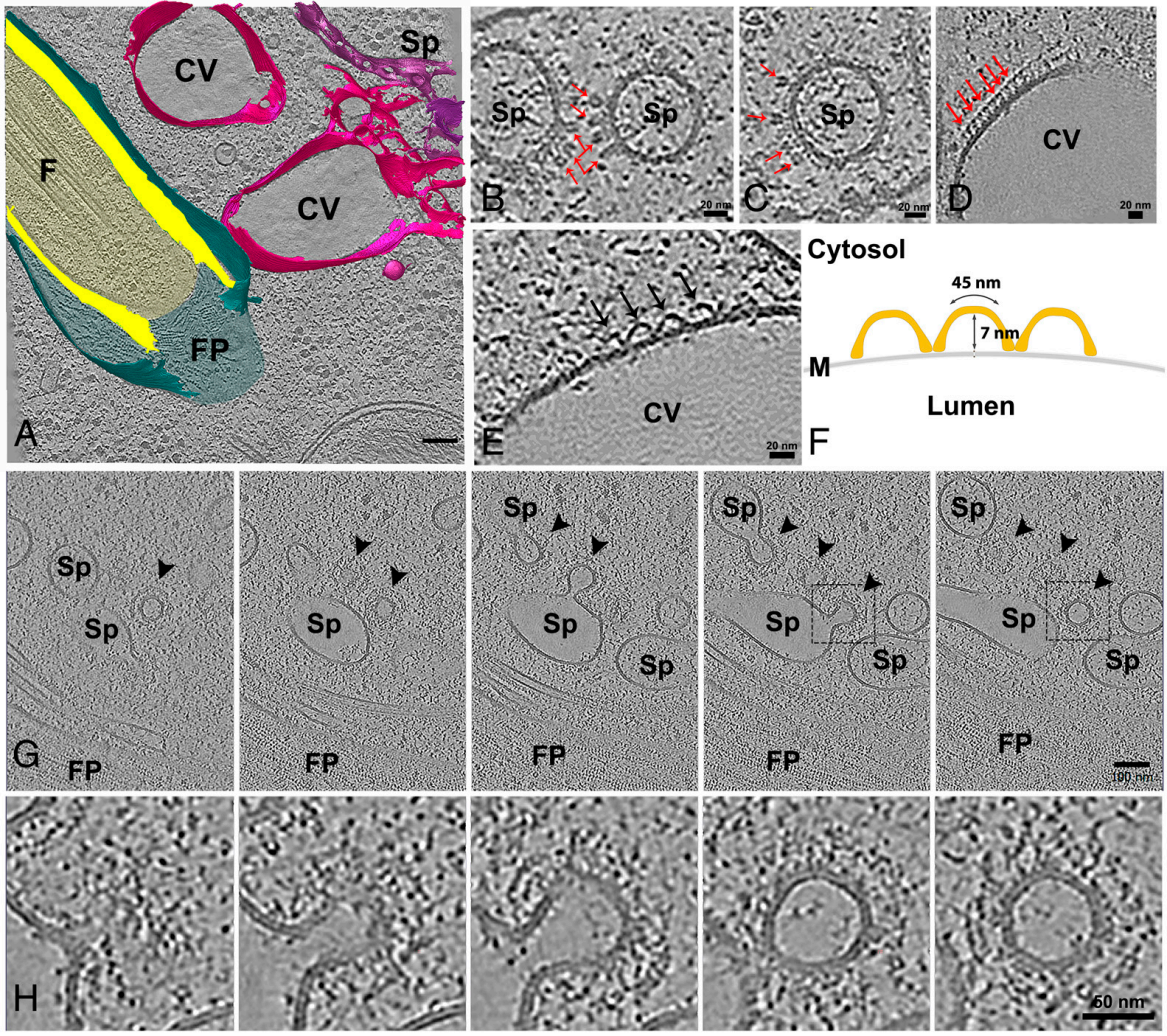
Structural organization of the CVC in *P. confusum*. (*A*) 3D reconstruction of a tomogram showing the FP (cyan) and two CV bladders (CV—pink) associated with the spongiome network (Sp—purple). ROIs are indicated in the reconstruction. (*B*–*D*) Tomogram slices showing the presence of spike-like proteins (red arrows) decorating the membranes of the spongiome (*B* and *C*) and the bladder (*D*). (*E*) A cross-sectional view of the CV bladder shows a region containing wavy-like protein scaffolds (black arrows). (*F*) Schematic representation of the wavy-like protein scaffolds showing the measured parameters, arc length and distance from top to membrane. (*G*) Sequential slices from the tomogram showing regions of the spongiome where the coated vesicle can be seen budding from the organelle. (*H*) Zoomed sequential slices through the vesicle indicated by the dashed rectangle in *G*.

**Fig. 5.**
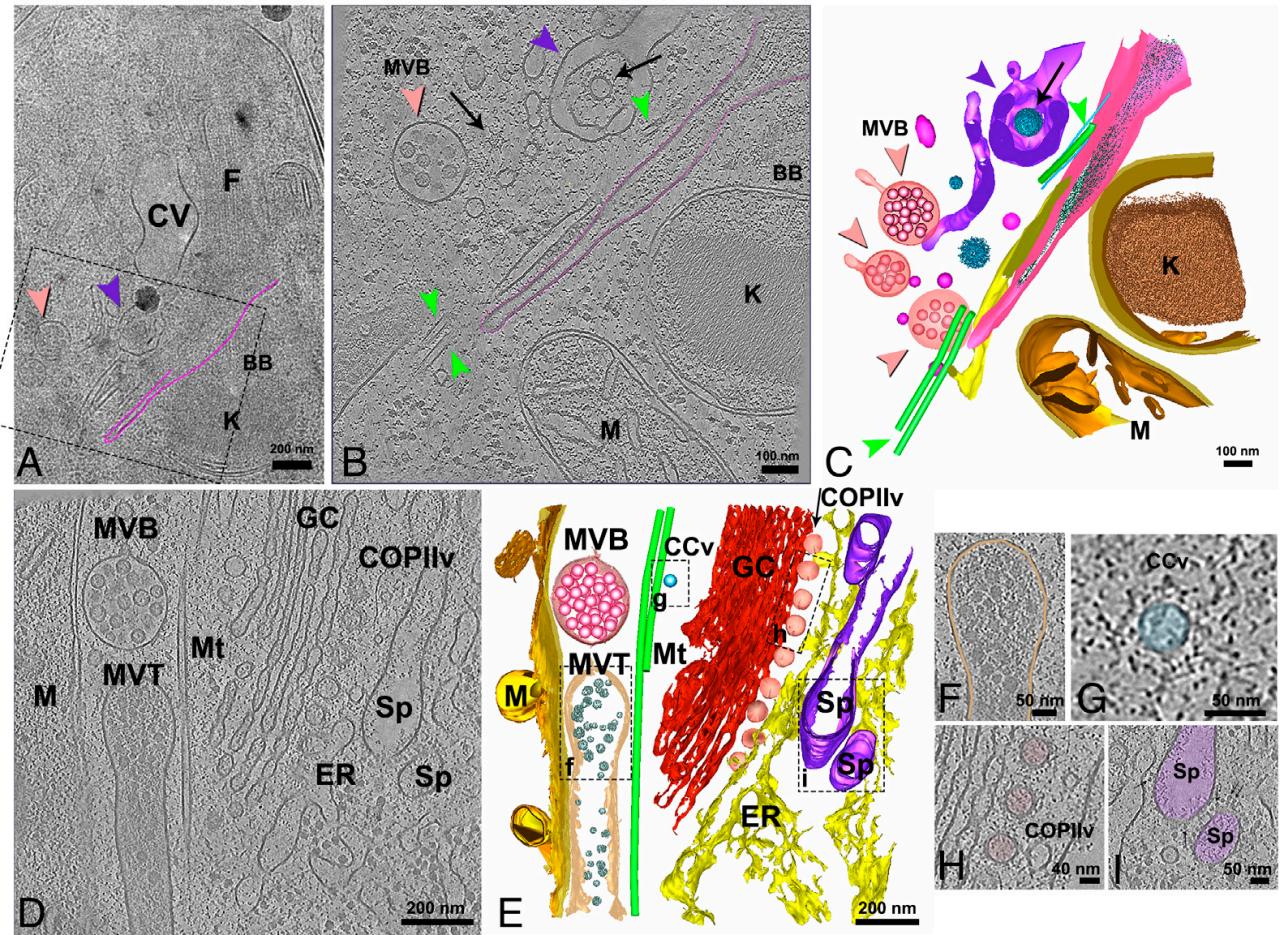
Structural organization of the biosynthetic-secretory-endocytic organelles. (*A*) Lamella map showing the region where the tomogram in (*B*) was acquired, encompassing the FP and adjacent structures, including the flagellum (F), CV, BB, and kinetoplast (K). The cytopharynx invagination is outlined in pink, and the boxed area marks the tomographic field. (*B*) Tomogram slice displaying the cytopharynx invagination, continuous with the FP membrane, as indicated by the presence of similar particles within both compartments. MTs (green arrowheads) line the cytopharynx. The spongiome (Sp; purple arrowhead) surrounds a coated vesicle (black arrow), and a MVB (salmon arrowhead) is visible nearby. (*C*) 3D segmentation of the same region showing the spatial arrangement of the cytopharynx (pink), FP particles (light green), MTs (green), spongiome (purple) with associated coated vesicles (blue), MVBs (salmon), and mitochondrion/kinetoplast (gold). (*D*) Tomogram from a more anterior region revealing organelles of the biosynthetic-secretory pathway, including the GC (red), ER (yellow), and coated vesicles (COPII-like; light pink) emerging from the ER toward the GC. Clathrin-coated vesicles (CCv; blue), MVBs (salmon), and a multivesicular tubule (MVT) containing dense vesicles are also present. (*E*) 3D model from the region in (*D*), illustrating the ER, GC, COPIIv, CCv, MVBs, MVT, Sp (purple), MTs (green), and mitochondrion (gold). (*F*–*I*) Virtual tomographic slices from the areas boxed in (*E*), highlighting ultrastructural details of (*F*) MVT; (*G*) CCv; (*H*) COPIIv; and (*I*) Sp tubules.

Another important feature observed in our tomograms were MT-associated elements (Fig. 2*E*). Some of these elements seemed to be composed of small dots of 3.7 nm of diameter and were associated parallelly with some of the FP MTs (Fig. 2 *E*, 1 and 2). We also observed a filament 9 nm-thick running parallel to a FP MT (Fig. 2 *E*, 3). A distinct set of filaments were also found to be associated with the FP exit site. These filaments (Fig. 2 *E*, 4 and 5) seemed to be associated with the FP membrane at the exit site and were linked to the collar filaments previously described. The filament shown in Fig. 2, 4 was composed of what seems to be 2 filaments 7 nm-thick each that turn around each other forming a rope-like structure. The filament shown in Fig. 2, 5, positioned posterior to Fig. 2 *E*, 4, had a diameter of 10 nm along its length.

The precise nature of these filaments remains uncertain, but their dimensions and associations suggest they may represent cytoskeletal elements with specialized functions near the FP. Notably, filamentous actin (F-actin) has never been visualized by electron microscopy in any trypanosomatid, including *T. brucei* and *T. cruzi*, despite the biochemical presence of actin being well established in these organisms (17). This longstanding absence of EM evidence has led to the assumption that trypanosomatid actin may not form canonical filaments, or that these filaments exist only transiently or in low abundance. However, the filaments observed here, particularly those forming rope-like or bundled morphologies, raise the possibility that under certain structural contexts, actin or actin-like proteins may polymerize into detectable structures. Further investigation, potentially combining cryo-ET with actin-specific labeling strategies, will be necessary to confirm their identity.

Interestingly, the *P. confusum* genome currently available in TriTrypDB includes annotated orthologs of actin 1, a conserved isoform across trypanosomatids, as well as actins 2 and 3, which have so far only been reported in *T. cruzi* and are absent from *T. brucei* and Leishmania spp. This suggests that *P. confusum* may possess a more diverse actin repertoire than previously anticipated. However, the genome also contains numerous entries annotated merely as “putative proteins,” including potential actin-binding and cytoskeletal components. This underscores the need for improved genome annotation and protein characterization to allow for more confident correlations between the observed filamentous structures and the underlying molecular machinery.

### Desmosome-Like Structures between FP Membrane and Flagellar Membrane.

Besides the cytoskeletal filaments of the FP collar and FP exit region, other structures were found. At the FP exit site, we observe proteins bridging the gap between FP membrane and the flagellar membrane (Fig. 3 *A*–*E*). These elements resembled desmosome junctions (Fig. 3*A*; see also *SI Appendix*, Figs. S4 and S5). At the cytosolic side, filaments attach to the FP membrane and appear to connect with the intermembrane bridges (Fig. 3 *C* and *F*). In some tomograms, we could see that these cytosolic filaments extend to the cytosol, far beyond the location where the desmosome-like structure is placed (*SI Appendix*, Fig. S4 *F* and *G*). From the flagellum side, two distinct plaques, present at the luminal side, are associated with an internal flagellar network of very thin filaments (2 nm thick). These filaments connect with the plaques and are directed toward the flagellum tip (Fig. 3 *C* and *F* and *SI Appendix*, Fig. S4 *B* and *C*). We noticed a preference for these filaments to associate with axonemal doublets 3 to 7 (Fig. 3 *A*–*C* and *SI Appendix*, Fig. S4 *A*, *D*, and *E*). In regions closer to flagellum exit from the FP, these filaments seem to intertwine perpendicularly forming a reticulated or braided structure. This organization suggests an orthogonal or mesh-like pattern, where individual filaments are arranged in a lattice-like configuration, which could indicate the beginning of the assembly of the paraflagellar rod (PFR) (*SI Appendix*, Fig. S4 *D* and *E*).

When looking at the structural details of the FP exit site in samples prepared for conventional TEM, we can see electron-dense plaques in this region, as shown in *SI Appendix*, Fig. S6. These structures correspond to the FAZ, previously described in *P. confusum* (2) and in *L. mexicana* promastigotes (15). However, with conventional TEM, what we mostly see are areas of increased electron density, without being able to distinguish their individual components. With cryo-ET, we were finally able to resolve the fine details of these structures, allowing us to see the individual elements that make up the FAZ, particularly the plaques on the flagellum side. This technique gives us a much clearer picture of how the FAZ is organized, something that was previously hidden by the limitations of traditional sample preparation methods.

### Other Structural Features At the Flagellum Exit Site.

The region of flagellum externalization was marked by distinct architectural features that appear to contribute to both flagellar anchoring and intracellular organization. Besides the already described desmosome-like structures, another striking observation was the presence of subpellicular MTs showing noncanonical morphologies as they approach the FP (*SI Appendix*, Figs. S5 and S7). These alterations, such as partial wall discontinuities and asymmetric cross-sections, were consistently observed in multiple tomograms from independent cells (n = 8). Although the exact number of protofilaments cannot be resolved under the present conditions, the data suggest a local transition in MT organization rather than an abrupt termination, as previously described in *T. cruzi* using conventional chemical fixation methods (14, 18–20). We cautiously describe this as a structural variation rather than a remodeling event, since the sequence of assembly or disassembly cannot be inferred from these static snapshots. Importantly, the use of cryo-FIB lamellae minimizes artifacts from sample preparation, and similar features were observed across multiple lamellae, reinforcing that they likely represent native structural states. Future analyses, including subtomogram averaging and complementary volumetric imaging, are required to better understand the molecular and architectural basis of these atypical MT configurations.

Adjacent to the FP membrane, we identified a thicker, specialized membrane domain flanked by spike-like protruding proteins extending into the cytosol (*SI Appendix*, Figs. S4 *D*–*F* and S5 *E* and *F*). This region is closely associated with a cytosolic density from which 3 MTs emerge, forming an organized array that extends toward the posterior region of the parasite. The spatial relationship between this membrane specialization and the MTs suggests that this structure may function as an anchor point for cytoskeletal organization, potentially playing a role in intracellular trafficking or cellular polarity.

### CV Complex Architecture.

The CV complex (CVC) is a membrane-bound organelle involved in osmoregulation, primarily found in freshwater protists, where it prevents excess water accumulation due to osmotic influx. This organelle is widely distributed across diverse eukaryotic lineages, including ciliates (e.g., Paramecium), amoebozoans (e.g., Dictyostelium), green algae (Chlamydomonas), and even some metazoans like freshwater sponges (21). In trypanosomatids, this organelle was identified in some species, but it is not universally present across the group. While *T. cruzi* (22, 23) and *Leptomonas collosoma* (24) possess a well-defined CVC, others, like *T. brucei*, appear to lack this organelle, suggesting differential retention or loss throughout evolution.

In this work, we report on the presence of a CVC in *P. confusum*. Our cryo-ET data revealed that *P. confusum* harbors two CV bladders, which are connected to an extensive network of tubules, collectively referred to as the spongiome (Fig. 4*A*). The bladders were positioned in close proximity to the FP membrane, a spatial arrangement that resembles what has been described in other trypanosomatids (22–24).

The spongiome tubules were decorated with spike-like protein structures, which appeared widely distributed across the tubule membranes (Fig. 4 *B* and *C*). Decorated tubules fused with the bladders, likely delivering spike-like proteins and other molecular components into the main vacuolar compartments (*SI Appendix*, Fig. S8), which also displayed those spike-like proteins at the surface (Fig. 4*D*). At the membrane of the main bladders, we observed a distinct wavy-like protein scaffold (Fig. 4*E* and *SI Appendix*, Fig. S8*A*). These scaffolds were not present at the membranes of the spongiome tubules. These protein scaffolds appear to periodically connect to the membrane, creating an arched conformation. Tomogram average allowed us to measure the arc length (~45 nm) and the maximum projection from the membrane (~7 nm) (Fig. 4*F*). Neither of these protein coats have been resolved before, not even in *T. cruzi*, where the ultrastructure of the CVC is the most extensively characterized (22, 23)

The structural features of the wavy-like scaffold strongly resemble those of the retromer complex, whose structure has already been deciphered using cryo-ET (25). The retromer is a key component in membrane remodeling and cargo sorting. This resemblance to retromer-like proteins supports the hypothesis that CVC could mediate dynamic membrane processes such as cargo sorting or vesicle trafficking, besides the osmoregulation. Indeed, in *T. cruzi*, proteins involved in vesicular traffic such as Rab11 and VAMP7 are localized to the CVC in this organism (26). Rab11 regulates the trafficking of glycosylphosphatidylinositol (GPI)-anchored proteins, including trans-sialidase, from the CVC to the cell surface (27).

The morphology of the spike-like proteins that decorated the membranes of the spongiome tubules and the bladders is similar with the structural organization of V-type proton ATPases (V-ATPases), as previously characterized by cryo-EM (28). Furthermore, the presence of V-ATPases has been extensively documented in the CVs of other protists (29–31) and was also identified as a component of the CVC in *T. cruzi* (26).

Another observed feature was the presence of several coated vesicles budding from the membrane of the spongiome tubules (Fig. 4 *G* and *H*). The coat around the vesicles looks similar to the organization of clathrin coated vesicles. In these contexts, clathrin has already been identified in the proteome of the CV of *T. cruzi* (26), but previous reports on the observation of clathrin coated vesicles budding from CVC are absent. Further analysis of subtomograms may clarify this point.

The localization of these proteins suggests that they play a role in proton transport and osmotic regulation, likely driving fluid uptake into the vacuole through active ion transport. The fact that these distinct protein structures coexist within the CVC suggests a functional division within the system and a role for the CV in intracellular membrane traffic. Further functional studies will be necessary to determine whether these features are unique to *P. confusum* or represent a conserved but previously overlooked aspect of CV organization in kinetoplastids.

### Endocytic and Biosynthetic-Secretory Organelles.

Stercorarian trypanosomatids are known to possess a cytostome–cytopharynx complex, a structure involved in the endocytosis of macromolecules from the extracellular environment (14, 32). Previous ultrastructural studies of *P. confusum* suggested that promastigote forms might also contain a cytostome–cytopharynx complex, based on scanning electron microscopy (SEM) observations of an indentation near the FP that appeared to resemble this endocytic structure (2).

However, in our CryoET analysis, which included 25 tomograms acquired from this region, we did not observe any evidence of a membrane invagination originating from the cell surface, as previously suggested. Instead, our data revealed a distinct structure: an invagination arising from the FP membrane itself, opening at the base of the FP (Fig. 5 *A*–*C* and *SI Appendix*, Fig. S10). This invagination, rather than being part of the cell surface, appears structurally integrated with the FP lumen, as we can observe FP luminal particles entering the beginning of the invagination but not filling it (Fig. 5*B*). This invagination was accompanied by a set of MTs that were longitudinally lining the structure. In our tomograms, we could count 3 MTs associated with the invagination (Fig. 5 *B* and *C* and *SI Appendix*, Fig. S10). Several multivesicular bodies (MVBs) and the tubules of the spongiome were seen in the vicinity of the FP and close to the invagination (Fig. 5 *B* and *C*).

The evolutionary implications of these findings are significant. The presence of a cytostome–cytopharynx complex opening from the FP membrane, as observed in *Trypanosoma raiae* (33), *Trypanosoma cyclops* (34) and *Trypanosoma lucknowi* (35), as well as in some bodonids (36) and euglenids (37), suggests that this organization may represent a more ancestral feature within kinetoplastids. In *T. cruzi*, the cytostome originates directly from the plasma membrane and extends into the cytopharynx (14). However, during cell division, the biogenesis of a new complex occurs from an invagination that opens inside the pocket and then is externalized at the end of cytokinesis (19). This demonstrates an alternative configuration in which endocytic structures remain structurally integrated with the FP. The occurrence of a FP-associated cytostome in both parasitic and free-living kinetoplastids (such as bodonids) raises interesting questions about the evolutionary pressures that led to the retention or loss of this structure. In free-living organisms like bodonids and euglenids, where phagotrophy plays a significant role in nutrient acquisition, a cytostome–cytopharynx complex opening from the FP may have facilitated efficient particle ingestion while maintaining a protective and controlled environment within the pocket. This configuration might have been co-opted by early parasitic trypanosomatids that relied on direct macromolecule uptake from their hosts, allowing for a transitional state between classical phagotrophy and the more specialized endocytic mechanisms seen in extant parasites. It´s important to highlight that *P. confusum* possesses all the genes for proteins already described in the cytostome–cytopharynx complex of *T. cruzi* (1, 38–40). However, a role in endocytosis in this parasite needs to be determined.

In the anterior region of the cell, near the cytopharynx invagination, we also observed organelles involved in the biosynthetic and secretory pathways (Fig. 5 *D*–*I*). These included a single GC, the interface region between the ER and Golgi, and coated vesicles emerging from the ER toward the Golgi apparatus (Fig. 5 *D* and *E*). The structure of these vesicle coats was reminiscent of COPII, suggesting they participate in anterograde transport (Fig. 5 *A* and *H*). From the trans-Golgi network, coated vesicles were observed, highlighting active sorting processes (Fig. 5 *D*–*G*). MVBs were commonly found adjacent to the GC (Fig. 5*D*). Additionally, we identified a tubular structure filled with electron-dense vesicles in proximity to the Golgi, which we termed the MVT, accompanied by cytosolic MTs running longitudinally along its length (Fig. 5 *D*–*F*). Tubules of the spongiome were also frequently observed near the Golgi and ER exit site, easily recognized by the presence of spike-like membrane proteins on their surface (Fig. 5 *D* and *I*).

**Fig. 6.**
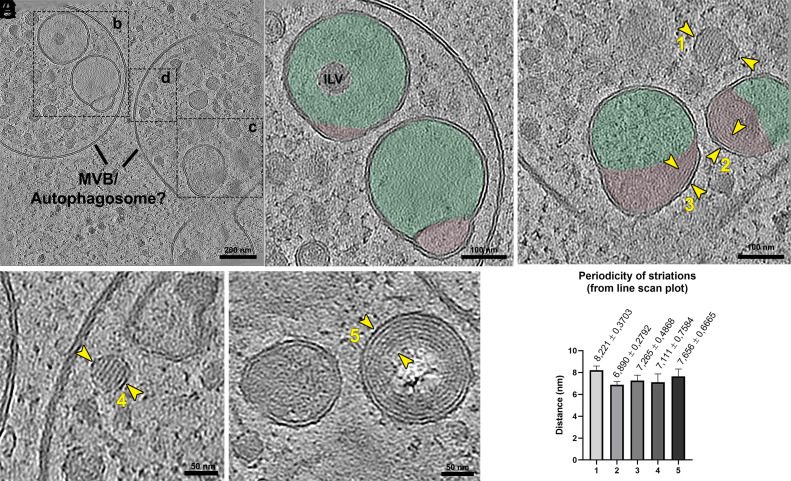
Ultrastructural features of autophagosome-like organelles in *P. confusum.* (*A*) Tomogram slice showing an overview of two large, rounded organelles containing multiple ILVs and larger hemifused vesicles. (*B*) Higher magnification view of the boxed region in (*A*), highlighting hemifused vesicles in which the larger vesicle (green) is connected to a smaller one (pink). Some of these larger vesicles also contain internal ILVs. (*C*) Another enlarged region from (*A*), showing hemifused vesicles with periodic striations along the membrane of the smaller vesicle (yellow arrowheads 2 and 3), and a small internal vesicle (arrowhead 1) also exhibiting internal striations. (*D*) Additional ILV containing striated patterns, highlighted between yellow arrowheads 4. (*E*) Multilamellar body showing striated patterns, with periodicity marked between yellow arrowheads 5. (*F*) Quantification of the spacing between striations in the five regions marked in panels (*C*–*E*), showing consistent periodicity across hemifused vesicles, ILVs, and multilamellar bodies. Mean spacing and SD are plotted for each region.

In the posterior region of the cell, we observed large, rounded organelles ranging from 800 nm to 1 µm in diameter, containing numerous intraluminal vesicles (ILVs) with diameters between 50 and 100 nm (Fig. 6*A* and *SI Appendix*, Fig. S11). Among these, we identified larger hemifused vesicles, composed of a major vesicle partially fused with a smaller one (Fig. 6 *A* and *B*). In some cases, the larger vesicle also enclosed additional ILVs. In other instances, hemifused vesicles displayed periodic striated patterns at the interface between the fused membranes (Fig. 6*C*). Similarly, other small vesicles within the organelle lumen exhibited periodic striation, as did multilamellar bodies located near the periphery (Fig. 6 *C*–*E*). We measured the periodicity of these striations across the different structures and found them to be remarkably consistent, ranging from 6.5 to 8.5 nm (Fig. 6*F*). Hemifused vesicles containing small lipidic bodies were also observed (*SI Appendix*, Fig. S11*B*).

These large MVBs resemble autolysosomes/autophagosomes based on their structural features, including the presence of multilamellar bodies and numerous ILVs. Notably, the hemifused vesicles observed within these compartments are morphologically identical to structures recently described in mammalian cells, by CryoET, and termed *hemifusomes* (41). These hemifusomes are proposed to represent precursors in the formation of ILVs through a mechanism distinct from the canonical ESCRT pathway. Their identification in *P. confusum* suggests that this alternative pathway may be evolutionarily conserved and present in ancestral eukaryotes. Particularly striking was the consistent presence of hemifusome-like vesicles within autophagosome-like compartments, hinting at a potential link between autophagy and noncanonical membrane remodeling mechanisms in early-branching eukaryotes. The periodic striations observed in the hemifused vesicles, ILVs, and multilamellar bodies are particularly intriguing. Similar striated patterns have been previously described as indicative of lipid crystalline phases in lipid droplets of mammalian cells (42). Comparable periodicity has also been reported in crystalline cholesterol-rich regions of lipid inclusions within the reservosomes of *T. cruzi* (43). The consistent spacing of these striations in *P. confusum* organelles—ranging from 6.5 to 8.5 nm—suggests a possible lipidic origin and points to conserved biophysical mechanisms underlying membrane organization and lipid storage across diverse eukaryotic lineages.

### Kinetoplast Architecture and Kinetoplast–BB Interactions.

The kinetoplast is a highly specialized region of the mitochondrion found in trypanosomatids, containing the most structurally complex mitochondrial DNA (kDNA) in nature. This network consists of thousands of circular DNA molecules—minicircles and maxicircles—that are topologically interlocked to form a dense structure (44). The kDNA topology is not uniform across trypanosomatids; it varies by species and even across different developmental forms within the same organism (45). These differences influence kDNA replication, segregation, and mitochondrial function. A key aspect of kinetoplast organization is its physical and functional interaction with the BB of the flagellum, mediated by the tripartite attachment complex (TAC) (46). This connection is crucial for coordinating kDNA segregation with cell division, ensuring accurate inheritance of the mitochondrial genome. Understanding these interactions provides valuable insight into the structural and evolutionary adaptations of kinetoplastids.

**Fig. 7.**
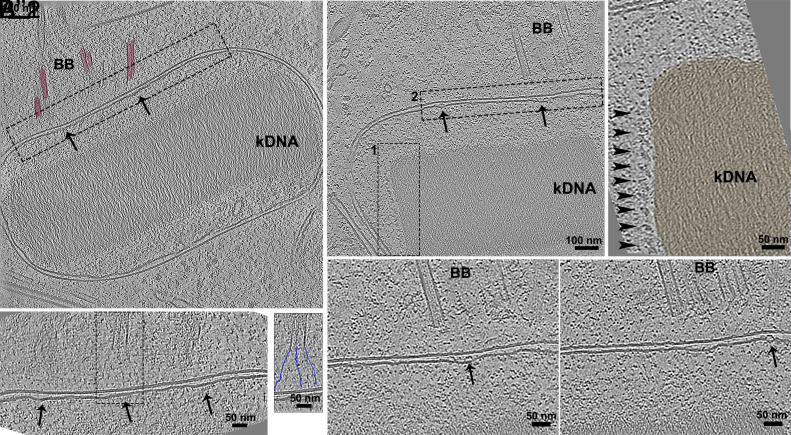
Organization of the kinetoplast and associated structures in *P. confusum*. (*A*) Cross-sectional slice of the kinetoplast showing a compact kDNA network with a longitudinal organization relative to the plane of the section. A dashed rectangle highlights the interface between the kinetoplast membrane and the BB (colored red), showing variations in curvature and thickness of the inner mitochondrial membrane. (*A*′) A higher magnification of the dashed rectangle in a showing a deformed inner membrane, particularly in regions near the BB MTs (black arrows). The rectangle highlights the fibers of the TAC, connecting the BB MT to the mitochondrial membrane. (*A*″) The *Inset* presents the segmented fibers in blue, providing a clearer visualization of this connection. (*B*) A different tomogram showing a view of the kinetoplast interface with the BB. ROI 1 marks the lateral side of the kDNA, where panel *B*′ 1 shows globular protein complexes associated with the lateral edge of the kDNA (arrowheads). ROI 2, shown in panel *B*′ 2, highlights the membrane interface between the kinetoplast and the BB. The two sequential tomogram slices in *B*′ 2 display bumped structures (arrows) associated with the inner mitochondrial membrane, located within the intermembrane space.

In *P. confusum*, kDNA was composed of tightly packed fibers with a disk-shaped morphology (Fig. 7). We observed that, while the outer membrane of the kinetoplast was smooth, the inner membrane exhibited deformations (Fig. 7*A′*) with “bumps” protruding toward the intermembrane space (Fig. 7 *B* and *B*′2). These were morphologically similar to the already characterized prohibitin protein scaffold found in higher eukaryotes (47) where it has a role as a scaffold for proteins and lipids regulating mitochondrial metabolism, including bioenergetics, biogenesis, and dynamics in order to determine the cell fate, death, or life. Prohibitins were shown to be essential for *T. brucei*, with knockdown leading to an altered mitochondrial morphology and decrease in mitochondrial membrane potential (48).

Faint fibers connect the BB to the kinetoplast outer membrane (Fig. 7*A*′), which may correspond to TAC. We could not identify any other components of the TAC (inner membrane-kDNA connections), probably due to the high noise levels and low contrast in our tomograms in comparison to conventional EM methodologies like deep-etching in combination with metal shadowing (49).

Additionally, we observed rolls of globular proteins aligned laterally at the kDNA (Fig. 7 *B* and *B*′1). These proteins probably are part of the antipodal sites that are macromolecular complexes involved in the kDNA duplication (44).

### Nuclear Structure: Nuclear Pore and Chromatin Organization.

**Fig. 8.**
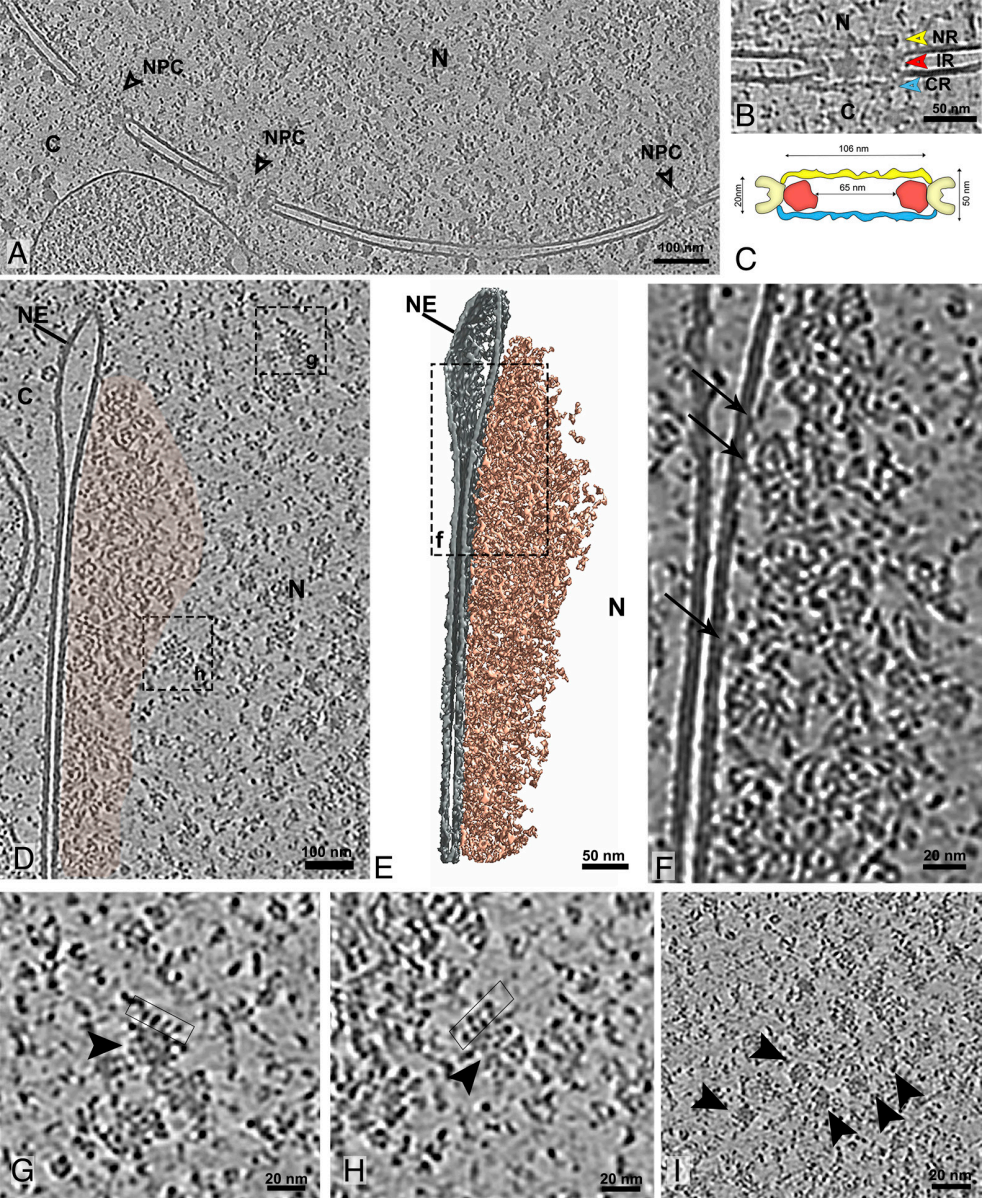
Nuclear organization of *P. confusum*. (*A*) Overview tomogram slice showing a portion of the nucleus with well-preserved nuclear envelope and embedded NPC. (*B*) Higher magnification of a single NPC highlighting its structural components: inner ring (red arrowhead), nuclear ring (yellow arrowhead), and cytosolic ring (CR, blue arrowhead). (*C*) Schematic representation of the nuclear pore, indicating average dimensions measured from tomograms. (*D*) Tomogram slice showing the nuclear envelope and associated chromatin (beige). (*E*) 3D segmentation of the region in (*D*), depicting chromatin filaments (beige) organized beneath the nuclear envelope. (*F*) Enlarged view of the boxed area in (*E*), showing filaments connecting the nuclear envelope to the chromatin network. (*F*) Zoom-in from the boxed region in (*D*), revealing filamentous structures resembling nucleosome chains (black arrowhead). (*H*) Enlarged view of another area from (*D*), displaying nucleosome-like filaments and looped structures (boxed). (*I*) Higher magnification showing macromolecular complexes scattered throughout the nuclear matrix (arrowhead).

Cryo-ET enabled high-resolution visualization of the nuclear envelope and nuclear pore complexes (NPCs) of *P. confusum*, which were remarkably well preserved. The nuclear envelope appeared as a continuous double membrane structure, and NPCs were frequently observed embedded along its surface (Fig. 8*A*). The morphology of the NPCs was consistent with that described in other eukaryotes, comprising a cytoplasmic ring, a central scaffold (inner ring), and a nuclear ring (Fig. 8*B*). These concentric structures framed a central channel spanning the nuclear envelope. Measurements of individual NPCs in our tomograms revealed an average pore diameter of ~106 nm. The width of the inner ring was approximately 20 nm, while the total distance between the cytoplasmic and nuclear rings was ~50 nm. The internal diameter of the central channel at the level of the inner ring was estimated to be ~65 nm (Fig. 8*C*). These dimensions closely match those reported in other unicellular eukaryotes (50), suggesting that the overall architecture of the NPC is evolutionarily conserved in kinetoplastids, including in this early-diverging lineage.

In addition to the NPC, cryo-ET revealed the intricate organization of chromatin in close association with the nuclear envelope (Fig. 8 *D* and *E*). Within the nuclear lumen, a network of fibers displaying looped structures resembling nucleosomes, similar to those previously visualized by cryo-ET in mammalian cells (51), was clearly observed (Fig. 8 *D*–*H*). These loops likely correspond to higher-order chromatin organization (Fig. 8 *G* and *H*). Scattered throughout the nucleoplasm, several macromolecular complexes were also detected, although their precise identity remains undetermined (Fig. 8*I*). Notably, we identified filamentous bridges connecting the inner nuclear membrane to the chromatin network (Fig. 8*F*). These structures may represent nuclear lamina-like elements previously described in other trypanosomatids (52), suggesting a conserved mechanism for chromatin tethering in this lineage.

A more extensive analysis of these regions through additional tomograms will be crucial to achieve a high-resolution structural characterization of the NPC and chromatin organization in *P. confusum*. In particular, subtomogram averaging could allow a detailed view of its individual subunits and symmetry features. Unfortunately, the limited number of tomograms acquired from this region in our dataset did not permit such an analysis. Future efforts focusing on this area may reveal specific adaptations or conserved structural elements of the NPC in this early-branching kinetoplastid.

### Glycosomes and Acidocalcisomes.

Acidocalcisomes are highly conserved organelles found across a wide range of organisms, from unicellular prokaryotes to multicellular eukaryotes (53). Their presence in such diverse lineages highlights their fundamental role in cellular homeostasis, particularly in ion storage, osmoregulation, and phosphate metabolism (54). Acidocalcisomes ultrastructure is highly impacted by the method of sample preparation. Conventional chemical fixation often led to distorted or partially empty acidocalcisomes, likely due to ion and molecule loss during the fixation and dehydration process. In contrast, high pressure freezing-freeze substitution (HPF-FS) methods preserved acidocalcisomes with a more compact and electron-dense appearance, revealing a well-defined internal organization (22).

**Fig. 9.**
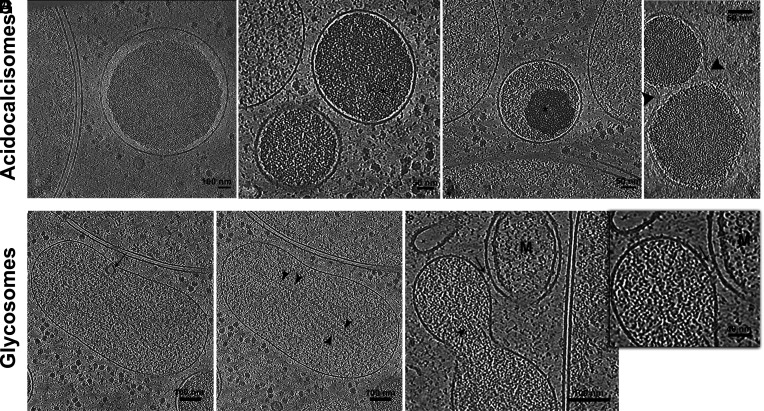
Structural organization of metabolic organelles, acidocalcisomes, and glycosomes. (*A*–*D*) Slices from different tomograms showing acidocalcisomes. (*A*) Acidocalcisome delineated by its membrane, followed by a cleared lumen and a centrally located, compact, electron-dense core. (*B*) Two acidocalcisomes are visible, both with a similar organization to panel (*A*), displaying a central electron-dense core. The upper acidocalcisome, contains a region within the compact material that is denser, indicated by an asterisk. (*C*) Acidocalcisome exhibiting two distinct regions of compact material: a less electron-dense region and a more electron-lucent zone closer to the organelle’s center. (*D*) Acidocalcisome undergoing apparent division, with a constricting membrane (indicated by an arrowhead) separating the electron-dense, compact material at the center of the organelle. (*E*–*G*) Slices from different tomograms showing glycosomes. (*E*) A larger glycosome containing a vesicle within its lumen, indicated by an arrow. (*F*) A different z-plane slice of the glycosome shown in panel (*E*), revealing an arrangement of filamentous proteins inside the organelle (arrowhead). (*G*) A glycosome appearing to be in division, also contains filamentous structures within its lumen (arrowhead) interacting with an adjacent mitochondrion (M). A dashed square highlights the region of interaction, and an *Inset* to the right shows a magnified view of this area, revealing a probable protein bridge connecting the glycosome membrane to the mitochondrial membrane (arrow).

The acidocalcisomes of *P. confusum* imaged with cryo-ET exhibit internal heterogeneity. Some contain a central, homogeneous electron-dense core, surrounded by a more translucent periphery (Fig. 9*A*), while others have a more heterogeneous core (Fig. 9 *B* and *C*). Organelles in fusion or fission showed a separated electron-dense core but a common constricted membrane (Fig. 9*D*). Our findings of different domains inside the acidocalcisomes are in accordance with what was previously shown by Girard-Dias et al. (55), who demonstrated that acidocalcisomes in *T. cruzi* contain distinct elemental nanodomains, forming segregated clusters within the organelle. This structural heterogeneity appears to be conserved across different trypanosomatid species, highlighting their functional importance in ion storage and homeostasis, suggesting that their substructural organization has been maintained as an adaptive strategy in diverse cellular environments.

Glycosomes are peroxisome-related organelles exclusive to kinetoplastids, where they play an essential role in metabolic compartmentalization (56). These organelles house most of the glycolytic enzymes, distinguishing them from peroxisomes in other eukaryotes. They exhibit an electron-dense matrix, characteristic for organelles that store and process metabolic enzymes, and have spherical or oval shapes (57).

Our analysis in *P. confusum* showed that glycosomes had a very dense matrix, compatible with previous findings by conventional EM (4). Most glycosomes were oval-shaped (Fig. 9*E*). Some glycosomes contained vesicles (Fig. 9*E*) or filamentous structures (Fig. 9 *E*–*G*) extending across the major axis of the organelle. The formation of filaments inside glycosomes may result from the polymerization of glycosomal enzymes, suggesting a potential mechanism for spatial organization within the organelle. Given the highly conserved nature of glycosomes across trypanosomatids, it will be essential to compare this structural feature in other species to determine whether filament formation is a unique characteristic of *P. confusum* or a broader, previously overlooked aspect of glycosome biology.

Contact sites between glycosomes and mitochondria were also observed (Fig. 9*G*) and are consistent with the metabolic role of glycosomes in energy metabolism.

## Conclusions

The application of cryo-ET to *P. confusum*, a basal trypanosomatid, has enabled the in situ visualization of cellular structures at an unprecedented level of detail in this evolutionary lineage. As a representative of the earliest branching group within trypanosomatids, *P. confusum* provides a critical window into the ancestral cellular architecture from which parasitic forms evolved. Our work demonstrates that cryo-FIB-SEM lamella preparation combined with high-resolution cryo-ET, is a powerful tool for uncovering structural features that are otherwise inaccessible using conventional methods.

Through this approach, we identified a repertoire of organelles and molecular assemblies, some of which were previously not previously documented in any trypanosomatid, including a cytopharynx-like invagination emerging from the FP, a bipartite CVC with distinct coats, and coated vesicles originating from both ER and Golgi, outlining a compartmentalized and dynamic biosynthetic-secretory pathway. The demonstration of clathrin budding/fusing with the spongiome tubules, MVBs, and a novel MVT indicates a complexity in vesicular trafficking that had not been previously appreciated in this group.

We revealed autophagosome-like compartments with hemifused vesicles and periodic lipid striations, structures strikingly similar to hemifusomes recently described in mammalian cells. Their presence in *P. confusum* suggests that alternative mechanisms of ILV formation, distinct from canonical ESCRT pathways, may have originated early in eukaryotic evolution and been conserved across distant lineages.

Finally, we described the architecture of the nuclear envelope and NPC, and described the organization of chromatin in nucleosome-like loops, including structures reminiscent of a nuclear lamina. These features not only underscore the structural sophistication of *P. confusum* but also support its value as a model to investigate the evolutionary emergence of cellular complexity in parasitic kinetoplastids.

In addition to its evolutionary relevance, *P. confusum* proved to be a well-behaved model organism for cryo-ET, allowing high-quality lamella preparation and cellular preservation. Further data acquisition will be essential to perform in situ high-resolution subtomogram averaging of the intriguing macromolecular structures observed throughout the cell.

By combining advanced imaging techniques with comparative analysis, this work opens the door to further exploration of the cellular innovations that define trypanosomatids. It serves as a reminder of how much we can still learn about these fascinating organisms and the evolutionary paths that shaped their biology.

## Materials and Methods

### Cell Culture.

*P. confusum* CUL13 (1) was obtained from the Biological Collection Bank from Fiocruz (Brazil). The parasites were axenically cultivated in RPMI medium supplemented with 10% FBS at 28 °C. 2 to 3 d cultures were used for experiments.

### Routine TEM.

Samples were prepared for TEM according to ref. 14. Thin-sections (60 to 80 nm thick) were observed in a Tecnai Spirit TEM (Thermo Fisher) operating at 120 kV.

### Cryosample Preparation and cryo-FIB Milling.

For cryo-EM experiments, cultures were first counted using a Neubauer chamber. A volume corresponding to 1.5 × 10^8^ cells was harvested by centrifugation (1,500×*g*, 5 min), and the resulting pellet was resuspended in 1 mL of fresh RPMI medium to reach a final density of 1.5 × 10^8^ cells/mL. From this suspension, 4 µL were applied to glow-discharged 200-mesh copper R2/1 holey carbon grids (Quantifoil Micro Tools GmbH, Jena, Germany). Grids were plunge-frozen in liquid ethane using a Vitrobot Mark 4 (Thermo Fisher Scientific, Waltham, MA). The blotting chamber conditions were set to 20 °C, 100% humidity, blot force 4 and a blot time of 5 s. The grids were blotted with a Teflon sheet from the side with the cells on the carbon film and a filter paper (Whatman No 1, Whatman, Maidstone, UK) from the reverse side. Grids were stored in liquid nitrogen until used.

For cryo-FIB milling, grids were clipped into cartridges with cutout (Thermo Fisher Scientific), mounted into a shuttle with 45° pretilt and transferred into a dual beam instrument with a scanning electron microscope (SEM) and Gallium focused ion beam (FIB) column (Aquilos 2, Thermo Fisher Scientific). The sample surface was sputter-coated with metallic platinum (t = 20 s, U = 1 kV, I = 10 mA, p = 0.2 mbar) and then coated with a layer of organometallic platinum using the gas injection system (GIS, Thermo Fisher Scientific) for 40 s, at the deposition position (stage tilt = 40°, stage rotation = 0°, z = 10.6 mm), before FIB-milling. The stage was then tilted to 25°, and trenches with a vertical distance of 10 µm were milled above and below a cluster of cells at an ion current of1 nA. Then, the stage was tilted to 15°, and the layer of cells thinned at 0.5 nA a thickness of 3 µm and at 0.1 nA to 1.2 µm. Afterward, the lamella was polished at 50 to 30 nA to its final thickness of 100 to 200 nm. For all milling steps, the FIB acceleration voltage was set to 30 kV. The progress of FIB-milling was monitored using the SEM operated at 3 kV and 13 pA.

### Cryo-ET.

Data were recorded in a Titan Krios G4 at 300 kV equipped with a Selectris X energy filter and a Falcon 4i camera (Thermo Fisher Scientific) using the Tomography 5 software package (version 5.12.0, Thermo Fisher Scientific). Lamella-overviews were recorded by stage-driven tiling at a nominal magnification of ×11,500 (pixel size, 2.127 nm). Tilt series were acquired at a nominal magnification of ×42,000 (pixel size 2.93 Å) or ×64,000 (pixel size 1.89 Å) and at each stage tilt the movie frames saved in EER file format. A dose-symmetric tilt scheme was used with an angular increment of 2° and a target defocus of −3 to −6 µm. Tilt series were collected in a tilt range of ±50° taking the lamella pretilt into account with a total dose of 100 to 125 e^−^/Å ^2^.

### Tomogram Reconstruction, Visualization, and Segmentation.

Data were processed using the TOMOMAN pipeline (version 0.6.9) (58). Fourteen frames with a dose of 0.14 to 0.18 e^−^/Å^2^ per frame were rendered from the EER frame stacks. These were used for motion correction in MotionCor2 version 1.4.7 (59) and CTF estimation with CTFFIND4 version 4.14 (60). Bad tilt images were removed after manual inspection and tilt-series dose filtered (61) using the respective TOMOMAN scripts. Tilt series were aligned with AreTomo (version 1.3.3) (62) and reconstructed with IMOD (63) (version 4.11.25) using the weighted back-projection algorithm.

For denoising, tilt series containing only the information of odd and even frames were obtained during motion correction, and subtomograms extracted from odd and even 4× binned tomograms used to train the neural network implemented in Cryo-CARE (64). Tomograms were visualized and cellular structures annotated in IMOD (63).

## Supplementary Material

Appendix 01 (PDF)

## Data Availability

All study data are included in the article and/or *SI Appendix*.
