# Supplementary material for: The subcellular architecture of Paratrypanosoma confusum revealed by CryoET: A window into early trypanosome evolution
Source: Proc Natl Acad Sci U S A. 2025 Dec 8;122(50):e2521233122. doi: 10.1073/pnas.2521233122 (PMC12718327; doi:10.1073/pnas.2521233122)
Supplement: Supplementary file 1 — Appendix 01 (PDF) [file pnas.2521233122.sapp.pdf]

Supporting Information for

The subcellular architecture of *Paratrypanosoma confusum* revealed by CryoET: A Window into Early Trypanosome Evolution

Carolina de Lima Alcantara[a], Matthias Pöge[d], Wolfgang Baumeister[d], Juergen Plitzko[e], Wanderley de Souza[a, b,c*]

[a] Instituto de Biofísica Carlos Chagas Filho, Universidade Federal do Rio de Janeiro, Rio de Janeiro, Rio de Janeiro, Brazil

[b] Centro Nacional de Biologia Estrutural e Bioimagem e Instituto Nacional de Ciência e Tecnologia em Biologia Estrutural e Bioimagens, Universidade Federal do Rio de Janeiro, Rio de Janeiro, Rio de Janeiro, Brazil

[c] Centro Multiusuário para Análise de Fenômenos Biomédicos, Universidade do Estado do Amazonas, Manaus, Brazil

[d] Research Group Molecular Structural Biology, Max Planck Institute of Biochemistry, Martinsried, Germany.

[e] Research Group CryoEM Technology, Max Planck Institute of Biochemistry, Martinsried, Germany

***Corresponding Author:** Wanderley de Souza

Email: wsouza@biof.ufrj.br

This PDF file includes:

Figures S1 to S11

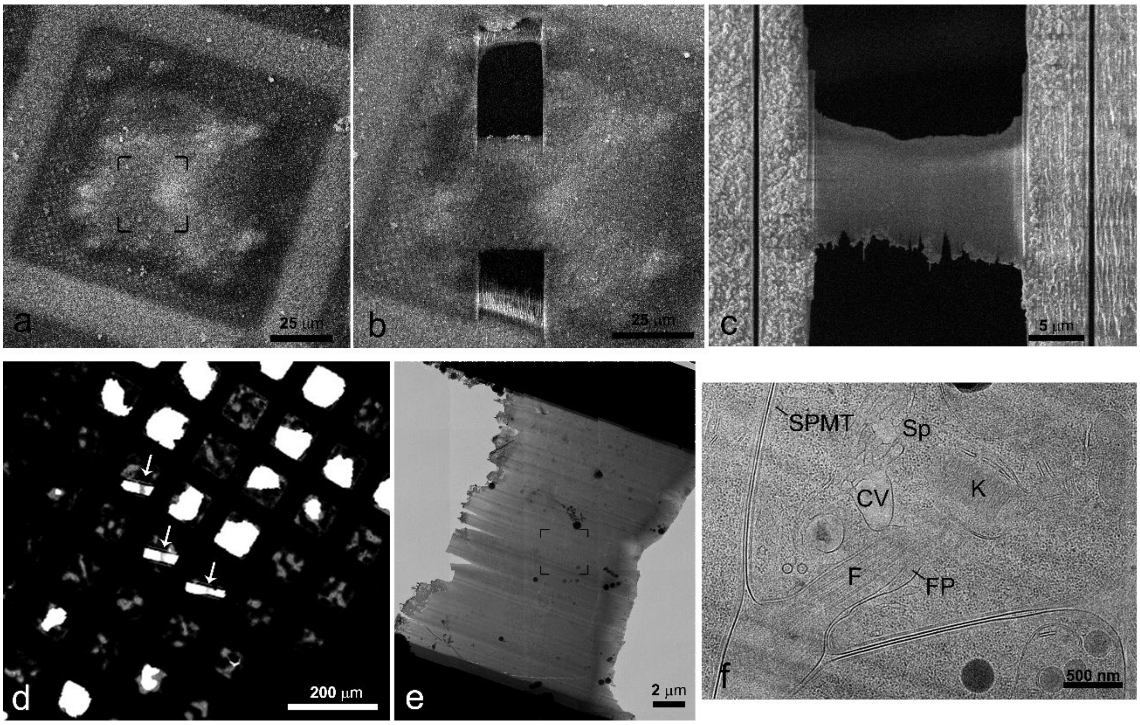

Supplementary Figure 1. Workflow and structural details of *Paratrypanosoma confusum* lamella preparation for cryo-electron tomography. (a) Scanning electron microscopy (SEM) image of a plunge-frozen grid showing the region of interest selected for lamella preparation. The marked square highlights the area used for focused ion beam (FIB) milling. **(b)** Higher magnification SEM image showing the initial milling of trenches around the region of interest. **(c)** SEM image of the final lamella after completion of the FIB milling process. **(d)** Transmission electron microscopy (TEM) image of the grid. White arrows indicate the positions of multiple lamellae produced in the previous steps. **(e)** Higher magnification TEM image of one lamella showing the area selected for tomogram acquisition, demarcated by a square. **(f)** High-resolution TEM image of the selected region of the *P. confusum* cell, highlighting key organelles and structures: CV (contractile vacuole), K (kinetoplast), Sp (spongiome), SPMT (subpellicular microtubules), FP (flagellar pocket), and F (flagellum).

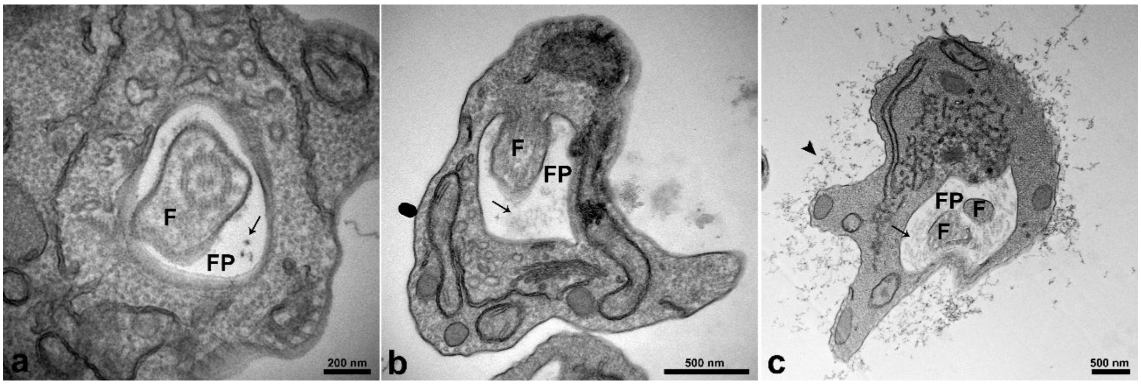

Supplementary figure 2. Flagellar pocket of *P. confusum* observed by conventional TEM. Micrographs of chemically fixed, dehydrated and heavy metal-stained cells showed the lumen of the flagellar pocket (FP) mostly empty (a), or with the presence of some particles and a low-density indistinguishable content (arrows). Some cells showed a highly dense surface coat composed of long fibrous material (arrowhead in c). F (flagellum).

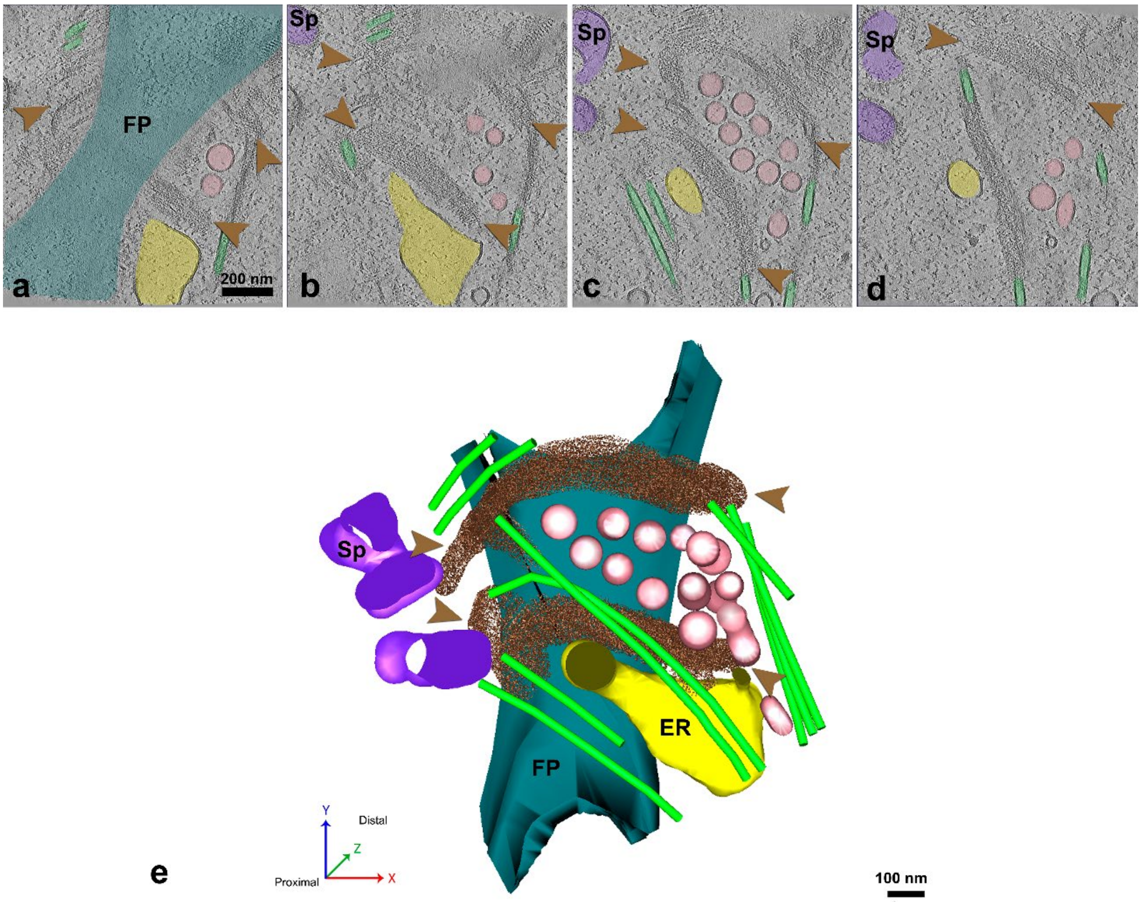

Supplementary figure 3. Cytoskeletal filaments and associated organelles around the flagellar pocket of *P. confusum*. (a-d) Sequential slices of a different tomogram from the one shown in Figure 4, illustrating the organization of the flagellar pocket and its associated structures. The flagellar pocket membrane is colored in moss green, while cytoskeletal filaments surrounding the flagellar pocket are indicated by brown arrowheads. Vesicles distributed among these filaments are colored in pink, and microtubules associated with the flagellar pocket are shown in green. A projection of the endoplasmic reticulum (ER) is colored in yellow. The images from panels (a) to (d) show the spatial arrangement of these filaments, revealing two distinct filament sets: one set proximal to the base of the flagellum, encircling the flagellar pocket neck region, and a second set more distal, positioned below the chain of vesicles. In some regions, the tubules of the spongiome, colored in purple, are also visible near the flagellar pocket. **(e)** 3D reconstruction of the tomogram, highlighting the spatial distribution of the structures around the flagellar pocket. Cytoskeletal filaments encircling the flagellar pocket, reconstructed through threshold segmentation, are shown in brown. The flagellar pocket membrane is depicted in moss green; microtubules are colored in green, vesicles in pink, and the spongiome tubules in purple.

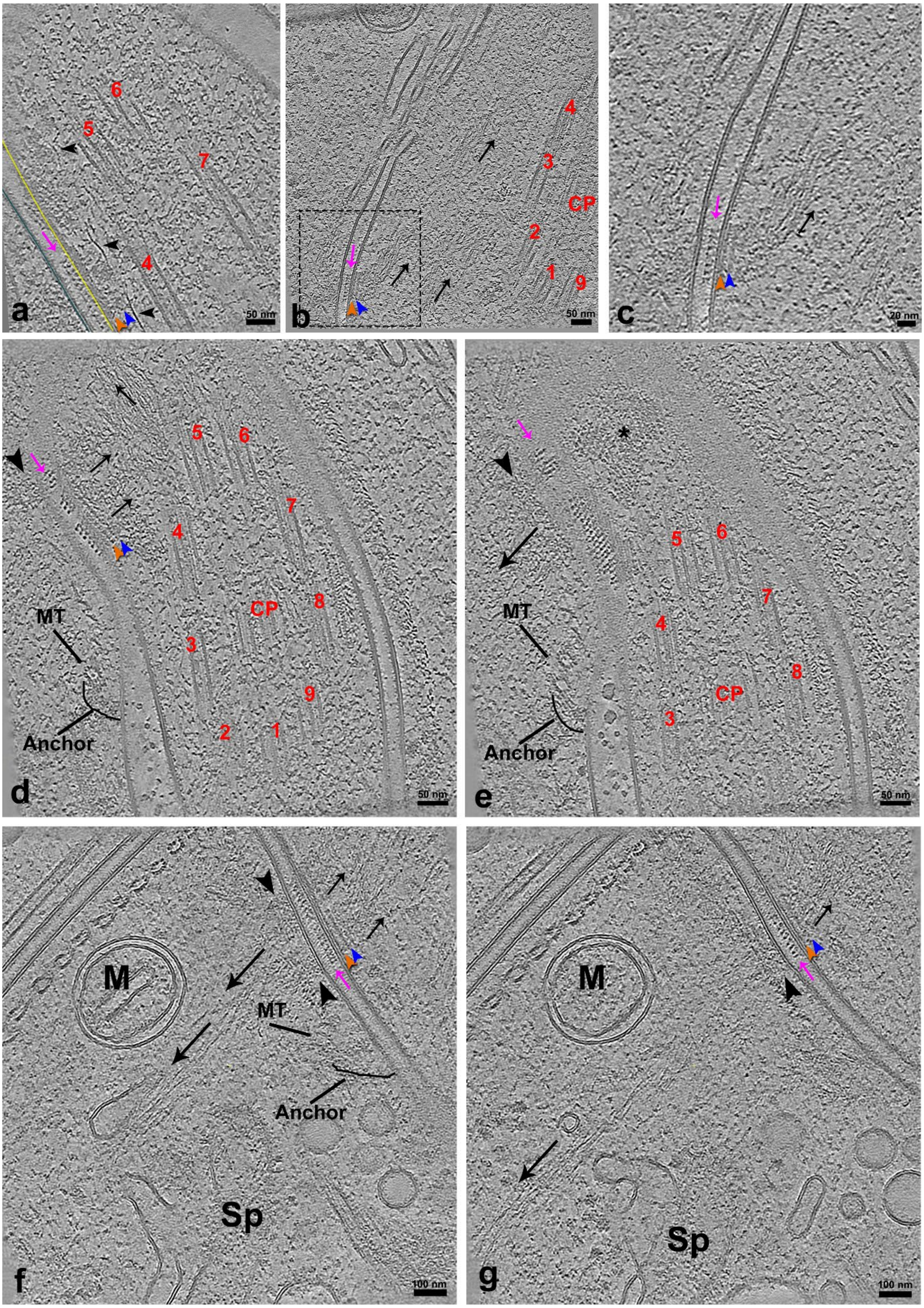

Supplementary figure 4. Ultrastructural organization of the desmosome-like proteins at the flagellum-flagellar pocket connection. Images are from different tomograms and show the bridges connecting the flagellar membrane to the flagellar

pocket membrane (pink arrow), the protein plaques at the flagellar side (orange and blue arrowheads) and the connection fibers at both sides (black arrow). (d,e,f) shows also the membrane domain at the flagellar pocket side, that forms an anchor e from where microtubules originates.

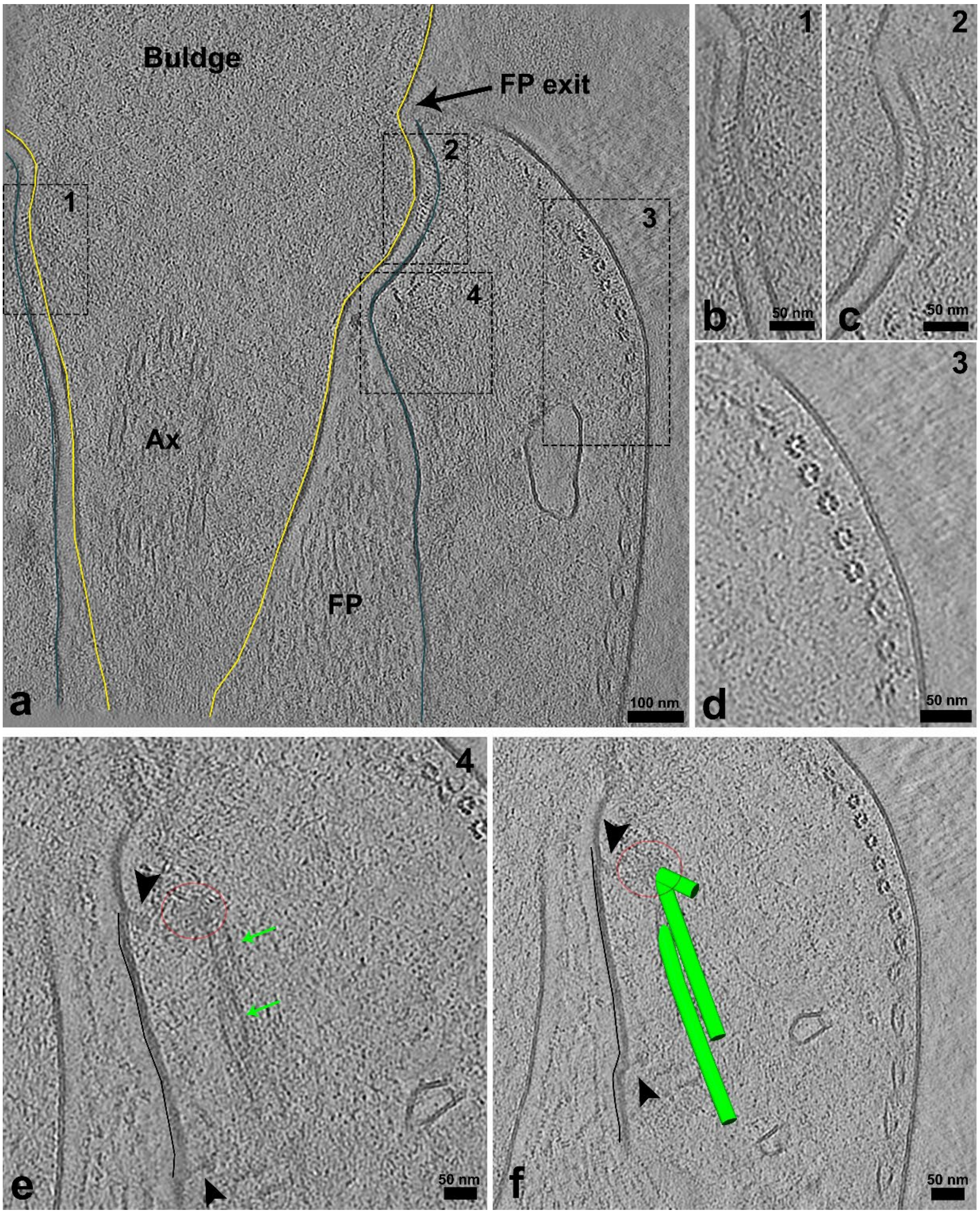

Supplementary figure 5. Membrane domains of the flagellar pocket and characteristics of subpellicular microtubules near the flagellum exit site in *P. confusum*. (a) Tomogram image of the flagellar pocket exit region, showing the extension of the flagellum outside of the pocket (buldge) and the flagellar axoneme (Ax). The flagellar membrane is outlined in yellow, and the FP membrane is outlined in green. The highlighted regions 1 and 2 show structures that connect the flagellar pocket membrane to the flagellar membrane, located near the flagellum exit. ROI 3 displays subpellicular microtubules near the flagellum exit, and ROI 4 reveals a structure associated with the flagellar pocket membrane from which some microtubules emerge. **(b,c)** Higher magnification views of ROIs 1 and 2 from panel (a), showing desmosome-

like connections between the FP membrane and the flagellar membrane around the flagellum exit site. **(d)** Image of ROI 3 from panel (a), depicting a reduction in the number of protofilaments in subpellicular microtubules as they approach the flagellar pocket near the flagellum exit. **(e)** Image of ROI 4 from panel (a), highlighting a differentiated, thicker region of the FP membrane (black line). This region is flanked by a set of protruding proteins extending from the flagellar pocket membrane (black arrowheads). A distinct cytosolic density is observed in this region (circled in red), from which microtubules emerge (green arrows). **(f)** Tomogram image of the same region as in panel (e), showing the emergence of microtubules from this distinct cytosolic density region, extending toward the posterior region of the parasite. This panel also shows the thicker region of the flagellar pocket membrane (outlined in black) flanked by the protruding proteins (black arrowheads), which extend from the membrane into the cytosol.

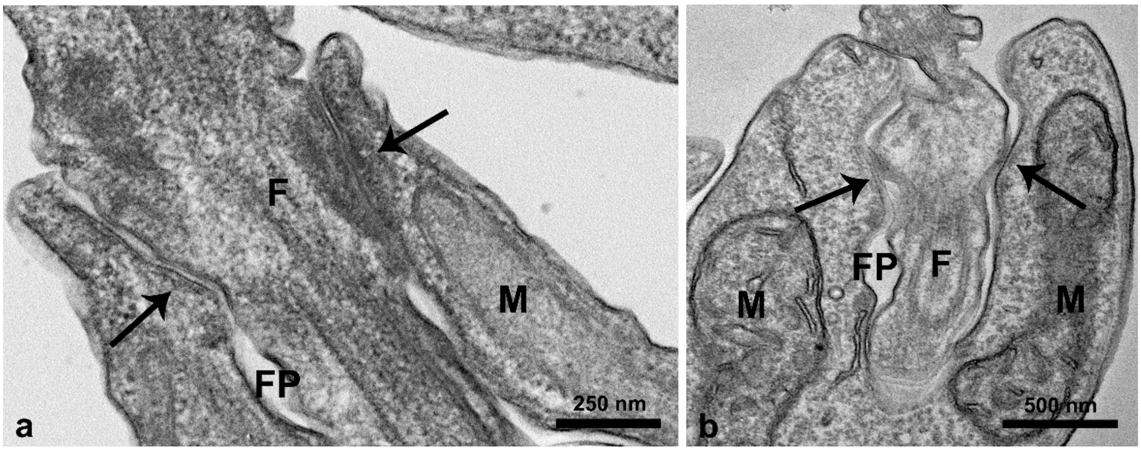

Supplementary figure 6. Resin-embedded TEM images of *P. confusum* showing membrane interactions near the flagellar exit from the flagellar pocket. **(a)** TEM image showing electron-dense regions at the interaction sites between the flagellar membrane and the flagellar pocket membrane, located near the flagellum exit. **(b)** Another TEM image showing similar proximity and electron density associated with the contact regions between the flagellar membrane and the flagellar pocket membrane, also near the flagellum exit.

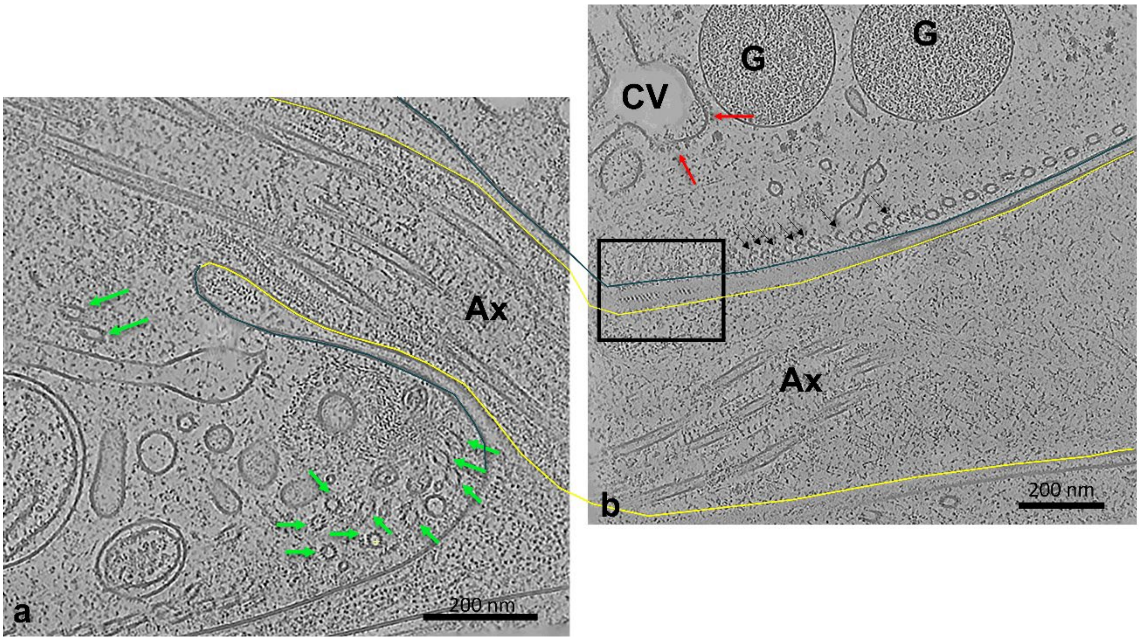

Supplementary figure 7. Montage tomograms of the region surrounding the flagellar pocket in *P. confusum*. (a) Tomogram showing the flagellar pocket exit region, with the flagellar pocket membrane outlined in dark green and the flagellar membrane outlined in yellow. Axoneme (Ax). In the cytosolic area near the flagellar exit, multiple microtubules are observed, some located close to the flagellar exit and others deeper within the cytosol (green arrows). **(b)** Continuation of the region shown in panel (a) in a different tomogram, displaying the contractile vacuole (CV) and associated spike-like proteins on the vacuole membrane (red arrows). Two glycosomes (G) are visible near the contractile vacuole, as well as the connection between the flagellar pocket membrane and the flagellar membrane, highlighted by a rectangle showing desmosome-like structures. The number of protofilaments in the subpellicular microtubules decreases as they approach the flagellar pocket, with microtubules showing fewer protofilaments (black arrows).

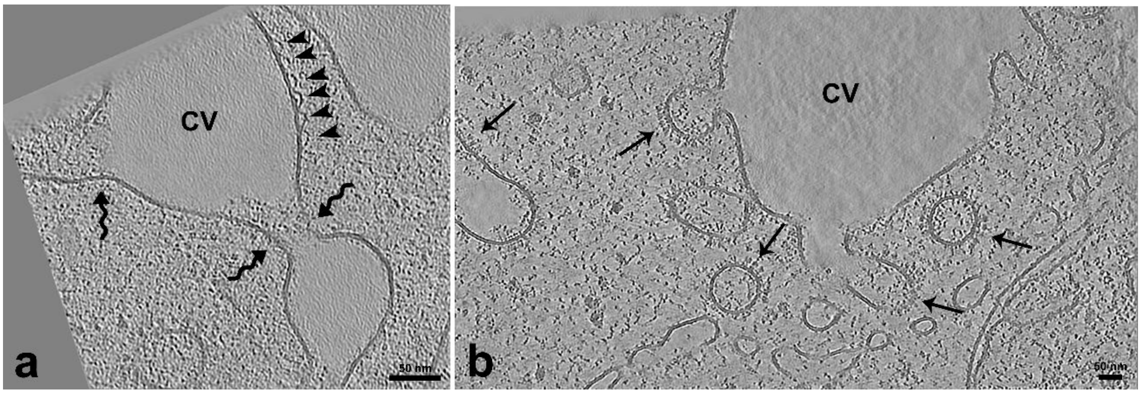

Supplementary Figure 8. Additional structural characteristics of the contractile vacuole complex in *P. confusum*, **(a)** Tomogram image from a different dataset than shown in Figure 7, rotated to highlight the regions of fusion between the main bladder of the contractile vacuole (CV) and the spongiome tubules. The wavy arrows indicate the fusion sites between the contractile vacuole bladder and the spongiome tubules. Scaffold proteins with a wavy pattern (arrowheads) are observed on the membrane of the main bladder of the CV. **(b)** Another tomogram showing additional regions of fusion between the CV and the spongiome tubules. Notably, the membranes of both the CV and the spongiome in the fusion areas are decorated with spike-like proteins, indicated by arrows.

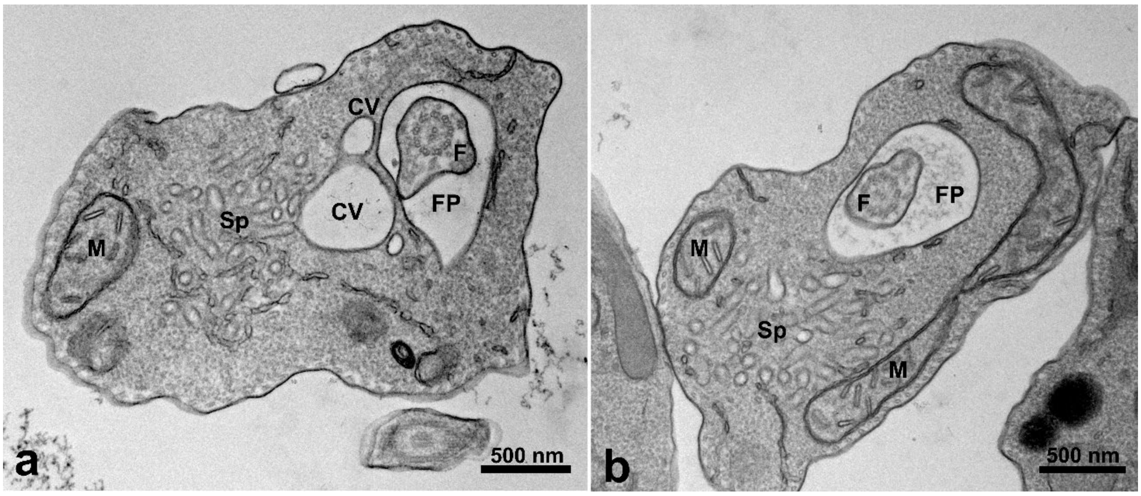

Supplementary Figure 9. Resin-embedded TEM images of *P. confusum* highlighting the contractile vacuole and associated structures. **(a)** Transverse section through the flagellar pocket (FP), showing the two bladders of the contractile vacuole complex and the associated spongiome tubules surrounding the area. **(b)** Another transverse section through the flagellar pocket (FP), displaying the presence of an extensive spongiome network with tubules occupying the regions near the flagellar pocket.

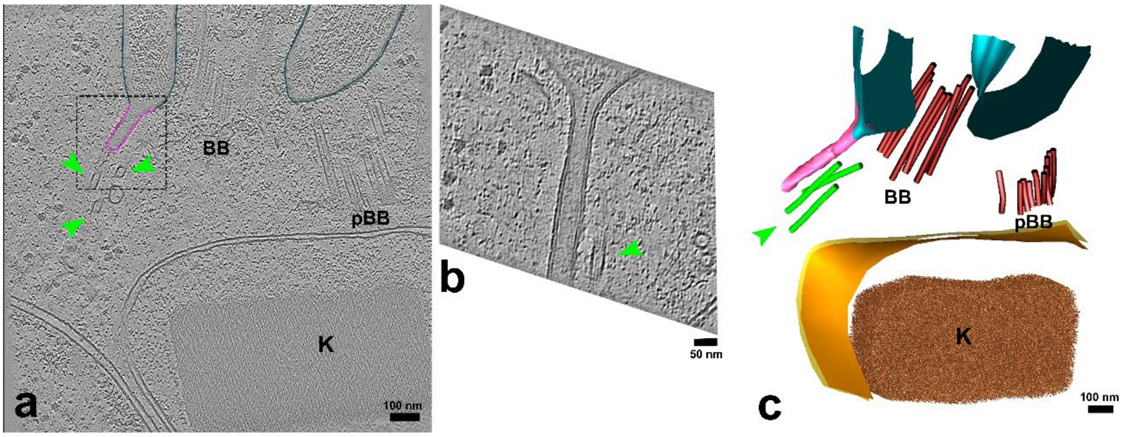

Supplementary Figure 10. Organization of the cytostome-cytopharynx complex in *P. confusum*. (a) Tomogram slice of a different tomogram acquired at the flagellar pocket vicinity, showing a membrane invagination at the base of the flagellar pocket. This invagination is accompanied by a set of three microtubules, marked by green arrowheads. The flagellar pocket membrane is outlined in cyan, and the invagination is delineated in pink. The boxed area indicates the region of interest. **(b)** Longitudinal view of the membrane invagination observed in panel (a), demonstrating the absence of particles inside the lumen of the invagination and showing the microtubules associated with it (green arrowhead). **(c)** 3D rendered image of the tomogram presented in panel (a), depicting the membrane invagination at the base of the flagellar pocket (pink) and its association with three microtubules (green). The FP is colored cyan. The basal body (BB) and probasal body (pBB) are shown in red. The kinetoplast (gold) is positioned just below the basal body.

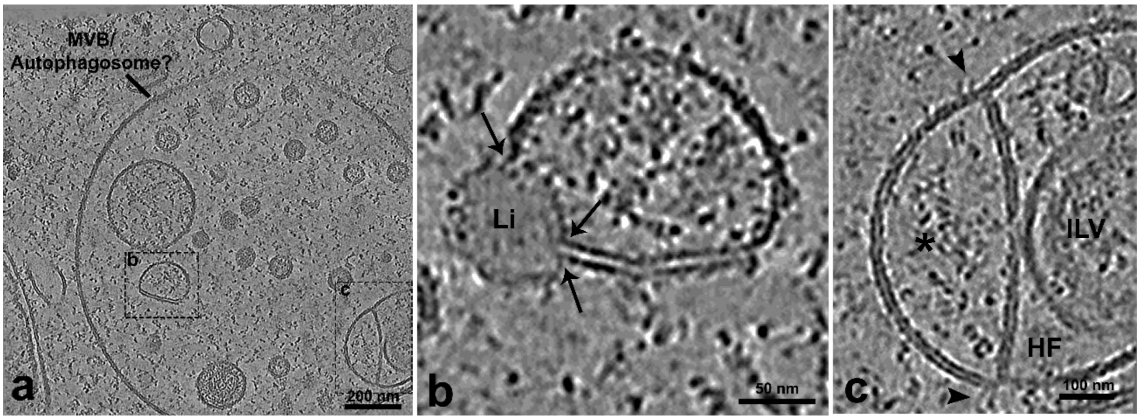

Supplementary Figure 11. Additional examples of autophagosome-like compartments and hemifused vesicles in *P. confusum*. (a) Tomographic slice from a different dataset showing a large, rounded organelle containing numerous intraluminal vesicles (ILVs), consistent with the autophagosome-like structures described in the main figure. (b) Higher magnification of the boxed region in (a), highlighting a lipid inclusion hemifused to a vesicle. Arrows indicate membrane continuity between the vesicle and the lipid body, revealing a hemifusion interface. (c) Close-up view of another hemifused vesicle pair. The larger vesicle contains ILVs, while the smaller one exhibits a heterogeneous internal content. At the interface between the two vesicles, small protrusions of unknown origin are observed on both sides of the fusion edge (arrowheads).
